# DNA interference is controlled by R-loop length in a type I-F1 CRISPR-Cas system

**DOI:** 10.1186/s12915-020-00799-z

**Published:** 2020-06-15

**Authors:** Donata Tuminauskaite, Danguole Norkunaite, Marija Fiodorovaite, Sarunas Tumas, Inga Songailiene, Giedre Tamulaitiene, Tomas Sinkunas

**Affiliations:** 1 Institute of Biotechnology, Life Sciences Center, Vilnius University, Sauletekio av. 7, LT-10257 Vilnius, Lithuania; 2 Pressent Address: Novo Nordisk Foundation Center for Biosustainability, Technical University of Denmark, 2800 Lyngby, Denmark

**Keywords:** CRISPR-Cas immunity, CRISPR protection, type I-F, Cascade, Csy, R-loop, Cas3 nuclease/helicase, Cas2/3, DNA interference, DNA degradation

## Abstract

**Background:**

CRISPR-Cas systems, which provide adaptive immunity against foreign nucleic acids in prokaryotes, can serve as useful molecular tools for multiple applications in genome engineering. Diverse CRISPR-Cas systems originating from distinct prokaryotes function through a common mechanism involving the assembly of small crRNA molecules and Cas proteins into a ribonucleoprotein (RNP) effector complex, and formation of an R-loop structure upon binding to the target DNA. Extensive research on the I-E subtype established the prototypical mechanism of DNA interference in type I systems, where the coordinated action of a ribonucleoprotein Cascade complex and Cas3 protein destroys foreign DNA. However, diverse protein composition between type I subtypes suggests differences in the mechanism of DNA interference that could be exploited for novel practical applications that call for further exploration of these systems.

**Results:**

Here we examined the mechanism of DNA interference provided by the type I-F1 system from *Aggregatibacter actinomycetemcomitans* D7S-1 (Aa). We show that functional Aa-Cascade complexes can be assembled not only with WT spacer of 32 nt but also with shorter or longer (14–176 nt) spacers. All complexes guided by the spacer bind to the target DNA sequence (protospacer) forming an R-loop when a C or CT protospacer adjacent motif (PAM) is present immediately upstream the protospacer (at −1 or −2,−1 position, respectively). The range of spacer and protospacer complementarity predetermine the length of the R-loop; however, only R-loops of WT length or longer trigger the nuclease/helicase Cas2/3, which initiates ATP-dependent unidirectional degradation at the PAM-distal end of the WT R-loop. Meanwhile, truncation of the WT R-loop at the PAM-distal end abolishes Cas2/3 cleavage.

**Conclusions:**

We provide a comprehensive characterisation of the DNA interference mechanism in the type I-F1 CRISPR-Cas system, which is different from the type I-E in a few aspects. First, DNA cleavage initiation, which usually happens at the PAM-proximal end in type I-E, is shifted to the PAM-distal end of WT R-loop in the type I-F1. Second, the R-loop length controls on/off switch of DNA interference in the type I-F1, while cleavage initiation is less restricted in the type I-E. These results indicate that DNA interference in type I-F1 systems is governed through a checkpoint provided by the Cascade complex, which verifies the appropriate length for the R-loop.

## Background

A constant evolutionary arms race between prokaryotic cells and their viruses (phages) leads to the development of diverse antiviral systems that are encoded by a substantial fraction of prokaryotic genes [1, 2]. One of the most widely spread defence systems is CRISPR (Clustered Regularly Interspaced Short Palindromic Repeats)-Cas (CRISPR-associated), which provide adaptive immunity against phage infections [3]. CRISPR locus functions as a repository, which stores information about cell intruders in a form of short DNA fragments called spacers that are surrounded by repetitive sequences termed repeats. Proteins encoded by *cas* genes are devoted to actuation of CRISPR-Cas function. The operation of CRISPR-Cas can be split into two modes: adaptation and effector, the latter can be further divided into (i) transcription and maturation and (ii) interference stages [3, 4]. The major players in the adaptation mode are an integrase Cas1 and a structural protein Cas2 that form a core of spacer integration complex [5]. The complex takes up DNA fragments of the intruder and inserts them into the CRISPR locus [6, 7]. In the effector mode, the transcript of the CRISPR region is cleaved within repeat sequences to produce small crRNA molecules, which together with Cas proteins assembles into a ribonucleoprotein (RNP) effector complex that detects and destroys invading nucleic acids in a crRNA-guided manner [8].

The diverse CRISPR-Cas systems are split into two classes according to the composition of the effector complex. The RNP complex composed of crRNA molecule and multiple Cas protein subunits is the characteristic of Class 1 systems that are further subdivided into types I, III, and IV [9]. One of the most numerous and widely spread CRISPR-Cas systems belong to the type I, which incorporates eight subtypes: A, B, C, G, D, E, F1 (previously F), F2 (previously F variant), and F3 [10, 11]. It was demonstrated that these systems can serve as useful molecular tools for genome engineering or modulation of metabolic pathways or even be applied in treatment from antibiotic-resistant bacterial infections [12–21]. Therefore, the huge potential might be hidden in this enormous variety, and mining of these systems might pave the way in new practical applications.

The interference stage in type I systems is coordinated by two components: Cas3 nuclease/helicase—a signature protein of the type I systems, and RNP complex named Cascade (CRISPR-associated complex for antiviral defence). Cascade complexes are assembled with similar overall structural architecture from crRNA and 3–5 different Cas proteins depending on the subtype [9, 22, 23]. The complex is responsible for targeting DNA that contains a spacer-matching sequence (protospacer) in near vicinity to additional motif termed PAM (protospacer adjacent motif) [24]. PAM sequence serves as a landing site for Cascade to check for the presence of protospacer [25–27]. When matching protospacer is detected, an R-loop structure begins to form where the spacer basepairs with the complementary strand of the protospacer in a zipper-like manner starting from the PAM, while the non-complementary strand is displaced as ssDNA [28–31]. When the spacer completely hybridises with the protospacer, the R-loop is locked signalling Cas3 to degrade target DNA [26, 32]. Cas3 grips the ssDNA strand in the locked R-loop and by hydrolysing ATP pulls this strand in a 3′ → 5′ direction and at the same time cuts it [33–36].

The molecular mechanism of DNA interference in type I is approximated from the most extensively studied type I-E systems. Although type I systems are evolutionarily related, their structural details differ. The closest relative of type I-E is the type I-F1 CRISPR-Cas system [11]. The type I-F1 is typified by a unique fusion of Cas2 to Cas3 (Cas2/3), which together with Cas1 form a 400-kDa complex with a Cas1_4_:Cas2/3_2_ stoichiometry [37]. The complex mediates spacer integration into CRISPR locus as demonstrated in *Pseudomonas aeruginosa* (Pae) and *Pectobacterium atrosepticum* (Pat) CRISPR-Cas systems [37–41]. Cas2/3 also degrades foreign DNA, which is targeted by the type I-F1 Cascade (elsewhere termed Csy) complex [39]. Cas proteins together with crRNA assemble into Cascade complexes with the similar overall structural arrangement in type I-E and I-F1: 5′-handle of the repeat is enfolded by the homologues of Cas5 and Cas7 proteins and Cas8 analogue; the spacer is bound by six Cas7 subunits forming the backbone of the Cascade; 3′-handle of the repeat is anchored by the homologue of Cas6. However, type I-F1 Cascade lacks small subunits Cas11 (previously termed Cse2), which form a “belly” of the type I-E Cascade and participate in R-loop locking, stabilising the displaced strand [32]. Furthermore, large subunits Cas8e and Cas8f1 have no structural similarity [23, 27, 32, 42–46]. The Cas8e (type I-E) recognises PAM and upon R-loop locking undergoes a conformational change that adjusts its surface for Cas3 positioning and loading to the displaced DNA strand of the R-loop [33]. Structural discrepancies between the subtypes imply that solutions for PAM sequence recognition, R-loop formation, Cas2/3 triggering, and target degradation might be different in the type I-F1.

Here we performed studies on interference stage of type I-F1 system from *Aggregatibacter actinomycetemcomitans* D7S-1 (Aa) bacteria. We show that Aa-CRISPR-Cas system interferes with target DNA in vivo and in vitro. Analysis of spacer length limits reveals that functional Aa-Cascade complex can be assembled with crRNA containing both truncated and elongated spacers. Upon binding to target DNA Aa-Cascade forms an R-loop, which length corresponds to the spacer length. Only the R-loop of WT spacer length (32 bp) or longer can engage the Cas2/3 nuclease/helicase, which degrades target DNA unidirectionally in an ATP-dependent manner. On the contrary, truncations of the WT R-loop at the PAM-distal end compromise Cas2/3-mediated DNA degradation. Our results reveal that DNA interference in the type I-F1 is governed by a checkpoint of the Cascade, which verifies the appropriate length for the R-loop. Taken together, we provide both similarities and differences between DNA interference mechanisms of the type I-F1 and I-E.

## Results

### Aa-CRISPR-Cas interferes with a DNA target containing promiscuous PAM in vivo

**Fig. 1:**
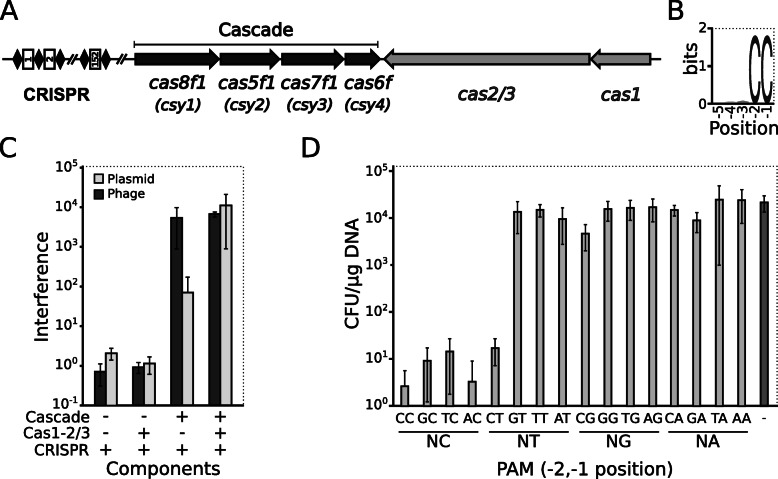
DNA interference of Aa-CRISPR-Cas system in vivo. **a** Schematic representation of the Aa-CRISPR-Cas locus composed of six *cas* genes and CRISPR locus containing 152 spacers. Cascade genes are underlined. Gene names of the previous nomenclature are in parentheses. **b** Predicted PAM for the Aa-CRISPR-Cas system. WebLogo representation [48] of 5-nt sequences found immediately upstream of phage protospacers that match CRISPR spacers. **c** Components of Aa-CRISPR-Cas that participate in plasmid or ssDNA phage interference. *E. coli* cells carrying (i) CRISPR, (ii) CRISPR and Cas1-Cas2/3 (Cas1–2/3), (iii) CRISPR and Cascade, or (iv) complete Aa-CRISPR-Cas system were transformed with pSP-CC plasmid or infected with M13-SP-CC phage that contain matching protospacer and CC PAM. The ratio of transformation or infection efficiencies of non-target and target DNA was expressed as interference. **d** Promiscuity of PAM in Aa-CRISPR-Cas system. *E. coli* cells bearing Aa-CRISPR-Cas system were transformed with a set of plasmids containing dinucleotide sequences adjacent to the spacer-matching protospacer and efficiency of transformation (CFU/μg) was assayed for each PAM variant. The plasmid containing a non-matching protospacer was used as a non-targeting control. Error bars in **c** and **d** represent standard deviations in at least three separate experiments (individual data values are provided in Additional file 14)

A human periodontal pathogen *A. actinomycetemcomitans* D7S-1 contains three Class 1 CRISPR-Cas systems: two of type I-C and one of type I-F1 [47]. Our focus was directed to the type I-F1 system because it carries a huge CRISPR locus of 152 spacers and thus could be potentially active at least in the adaptation stage. The CRISPR region is separated from six type I-F1 *cas* genes by about 253 kbp. These genes are arranged into a *cas8f1-cas5f1-cas7f1-cas6f* cluster that encodes proteins of the Cascade and a cassette of *cas1-cas2/3*, which encodes a Cas1 DNA integrase and a fusion protein of Cas2 and Cas3 nuclease/helicase (Cas2/3) (Fig. 1a). The most common spacer length in the CRISPR locus is 32 bp, although few spacers of 31, 33, and 56 bp length could be found as well (Additional file 1: Table S1). We performed a sequence similarity search for all spacers and found 13 perfectly matching protospacer sequences in the genomes of two *A. actinomycetemcomitans* phages. By clustering surrounding sequences of the protospacers, we identified a 2-bp conserved motif 5′-CC-3′ immediately upstream of the protospacers (Fig. 1b and Additional file 1: Table S1), which may correspond to a putative PAM sequence of this system.

To investigate if the type I-F1 Aa-CRISPR-Cas system can interfere with a foreign DNA, we transformed *Escherichia coli* with expression vectors bearing Cascade cluster, *cas1-cas2/3* genes and CRISPR region that contained only one repeat-spacer-repeat unit (the leader sequence was omitted to avoid the influence of adaptation mode) or respective empty vector. Target sequence containing putative CC PAM in the vicinity to the matching protospacer was cloned into a pUC19 plasmid and M13mp18 phage DNA. The target DNA was then inserted into *E. coli* cells, which carried all or some components of the recombinant Aa-CRISPR-Cas system, and transformation or infection efficiency of plasmid or phage DNA, respectively, was monitored. We show that expression of all Cas proteins and CRISPR region of the Aa-CRISPR-Cas system was required to efficiently interfere with dsDNA of multi-copy plasmid, although interference of the plasmid even in the context of the only Cascade-forming components was markedly increased. On the other hand, Cascade complex alone was sufficient to suppress infection of ssDNA phage. As anticipated, the expression of the CRISPR region alone or together with Cas1-Cas2/3 proteins did not protect against the DNA targets (Fig. 1c).

PAM sequences from different type I-E CRISPR-Cas systems are diverse and promiscuous [34, 49], while CC PAM sequence is characteristic for different type I-F systems [41, 50]. To examine if CC PAM is the unique sequence recognised by Aa-CRISPR-Cas system, we assayed transformation efficiency of a set of DNA targets containing different dinucleotide sequences at −2 and −1 positions upstream of the protospacer. We show that 5′-NC-3′ (where N is C, T, G, or A) and 5′-CT-3′ dinucleotides can serve as the PAMs (Fig. 1d).

### Aa-CRISPR-Cas interferes with dsDNA in vitro

**Fig. 2:**
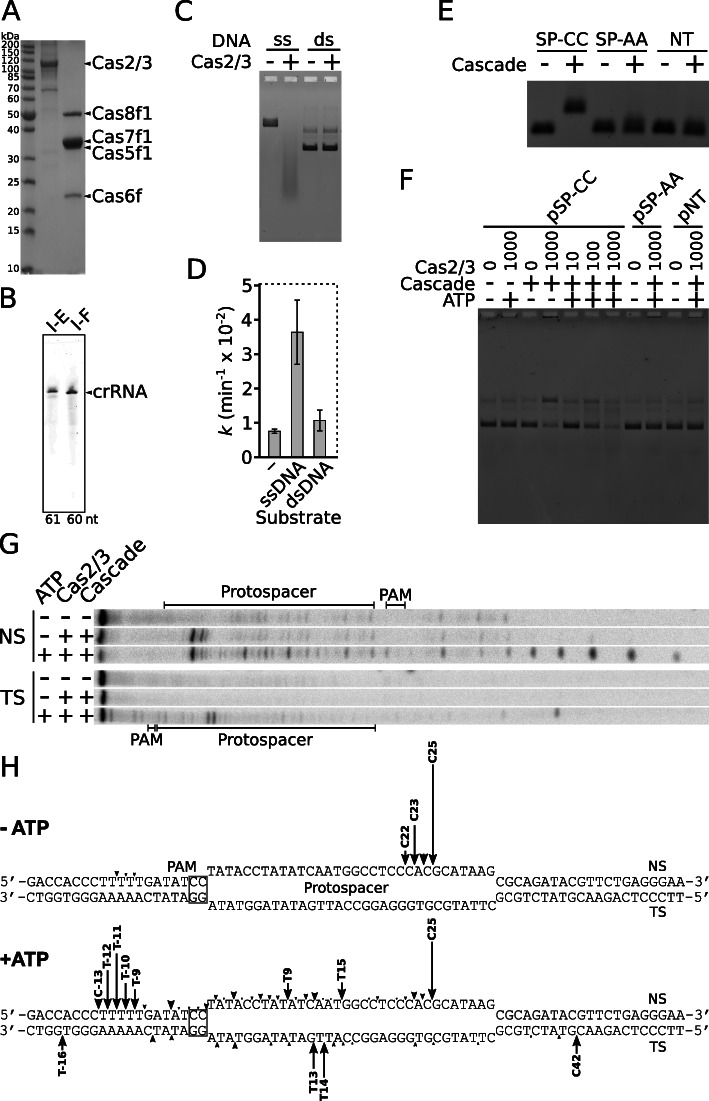
Reconstitution of DNA interference of the Aa-CRISPR-Cas system in vitro. **a** SDS-PAGE of purified Cas2/3 protein and WT Cascade complex preparations. Expected molecular weights for Cas2/3-His and Cascade-forming Cas8f1, Cas5f1, Cas7f1, and His-Cas6f proteins are approximately 127, 52, 36, 38, and 23 kDa, respectively. **b** crRNA of the type I-E *S. thermophilus* Cascade complex is shown for molecular weight comparison (lane I-E). Length of the respective crRNA is indicated below the gel. **c** ssDNA nuclease activity of Cas2/3. Cas2/3 nuclease activity assayed using M13mp18 (ssDNA) or pSP-CC (dsDNA) as substrates. **d** ATP hydrolysis by Cas2/3. Reaction rate constant *k* values (min^−1^) of ATP hydrolysis by Cas2/3 were monitored in the presence of M13mp18 (ssDNA), pSP-CC (dsDNA), or in the absence of DNA (−). Error bars represent standard deviations for at least three separate experiments (individual data values are provided in Additional file 14). **e** DNA binding by Cascade. WT Cascade interaction with target DNA (SP-CC) bearing CC PAM and matching protospacer or non-target DNA containing defective AA PAM (SP-AA) or no matching sequence (NT) was assayed by EMSA in agarose gel. **f** Cascade-targeted dsDNA degradation by Cas2/3. Degradation of respective plasmids in the presence (+) or absence (−) of ATP and Cascade was initiated by addition of Cas2/3. **g** Cas2/3 cleavage within the R-loop. SP-CC oligoduplex ^32^P-5′-labelled on either the non-target (NS) or target (TS) DNA strand was incubated with WT Cascade and Cas2/3 in the absence (−) or presence (+) of ATP. **h** Map of Cas2/3 cleavage sites. Arrows indicate cleavage positions within SP-CC sequence. The height of the arrow correlates with a relative amount of cleavage product (for more details see Additional file 5: Figure S4)

Type I CRISPR-Cas systems destroy DNA by the coordinated action of two components: Cascade complex, which is responsible for target DNA recognition, and Cas3 nuclease/helicase that degrades the Cascade-targeted DNA. To verify the molecular mechanism of Aa-CRISPR-Cas in vitro, we first expressed and purified the recombinant Cascade complex and Cas2/3 protein. As expected, the purified ribonucleoprotein Cascade complex is composed of Cas8f1, Cas5f1, Cas7f1, and Cas6f proteins (Fig. 2a), which are bound to a crRNA molecule (Fig. 2b). Then we assayed biochemical activities of separate components of Aa-CRISPR-Cas interference machinery and afterwards reconstituted DNA interference stage in vitro.

The Cas2/3 protein is composed of Cas2, HD nuclease, and helicase domains [38]. We show that Cas2/3 degrades ssDNA non-specifically leaving dsDNA intact (Fig. 2c). The nuclease activity is supported by Co^2+^, Mn^2+^, and Ni^2+^ divalent metal ions (Additional file 2: Figure S1A). Similarly to the type I-E Cas3 protein, Cas2/3 protein possesses an ssDNA-stimulated ATPase activity, which remains at the basal level in the presence of dsDNA (Fig. 2d). The ATP hydrolysis is promoted by Mg^2+^, Ca^2+^, Mn^2+^, and Co^2+^ divalent metal ions (Additional file 2: Figure S1B).

Cascade complexes are responsible for surveillance and binding to the target DNA upon its recognition [26]. We performed EMSA analysis of Aa-Cascade binding to three dsDNA substrates: (i) containing PAM and complementary protospacer (SP-CC), (ii) containing protospacer without PAM (SP-AA), and (iii) lacking both PAM and protospacer (NT). We show that Aa-Cascade binds only to DNA target SP-CC, which contains PAM and complementary protospacer. DNA substrates SP-AA and NT were not bound by the Cascade (Fig. 2e). Next, we analysed an R-loop formation by employing footprint assay with P1 nuclease. The displaced ssDNA is susceptible to P1 nuclease degradation (18th–25th nt from the PAM), while the DNA-RNA hybrid is intact (Additional file 3: Figure S2). We show that Cascade binding to the DNA target leads to R-loop formation where the spacer basepairs with the complementary strand of the protospacer, while the non-complementary DNA strand is displaced in the ssDNA form.

We assembled Aa-Cascade and Cas2/3 with a set of dsDNA targets and reconstituted interference stage in vitro. We used three supercoiled plasmid DNAs as substrates: (i) containing CC PAM and complementary protospacer (pSP-CC); (ii) containing protospacer, but lacking PAM (pSP-AA); and (iii) lacking both PAM and protospacer (pNT). The plasmid pSP-CC was cleaved only in the presence of both Cascade and Cas2/3. In the absence of ATP, nicked DNA product was accumulating, while ATP promoted degradation of dsDNA target. Neither nicking nor degradation was observed for the supercoiled plasmids pSP-AA or pNT (Fig. 2f).

To determine the directionality of Cas2/3 degradation, we utilised a set of linear dsDNA substrates with varying positions of the PAM-protospacer according to the DNA ends. The substrates were obtained by digestion of pSP-CC plasmid with different restriction endonucleases (Additional file 4: Figure S3A). Cleavage profiles of these linear substrates after treatment with Cascade and Cas2/3 revealed unidirectional degradation upstream from the protospacer leaving downstream sequence intact (Additional file 4: Figure S3B). Next, precise cleavage positions of Cas2/3 were determined using ^32^P-labelled DNA oligoduplex in either the non-target or target strand (Fig. 2g and Additional file 5: Figure S4). In the absence of ATP, only the displaced DNA strand in the R-loop is hydrolysed between 22nd and 25th positions from the PAM. In the presence of ATP, the non-target strand is cleaved at multiple positions upstream from the 25th position of the protospacer, while the major hydrolysis products of the target strand are positioned within the protospacer (Fig. 2h and Additional file 5: Figure S4).

### The Aa-Cascade complex can incorporate crRNA with varying spacer length

**Fig. 3:**
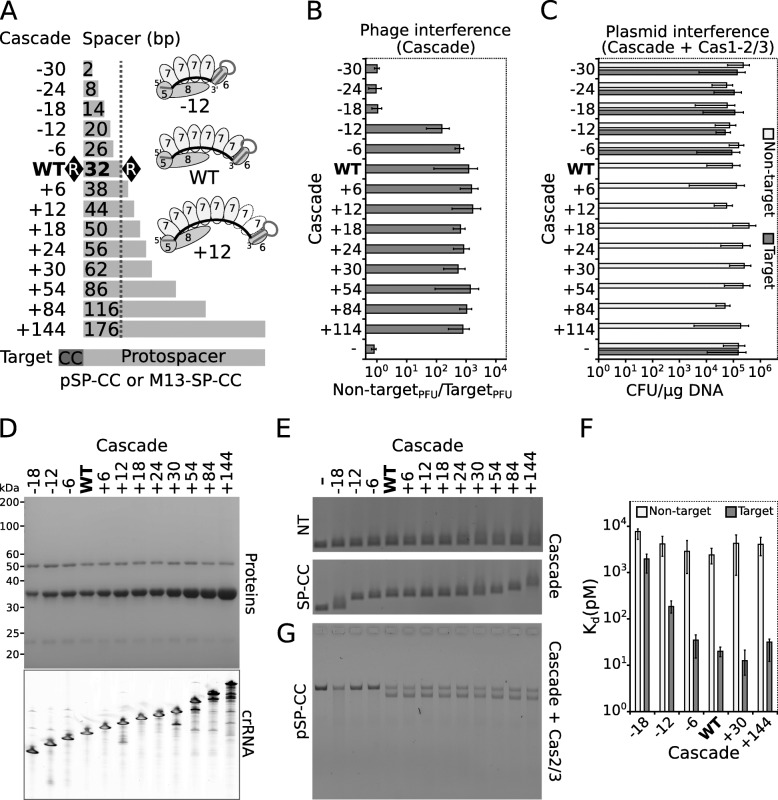
Influence of spacer length on DNA interference. **a** Constructs of altered spacer length. WT Cascade contains spacer of 32 nt length, which was truncated or extended by increments of 6 nt at the spacer’s 3′-end. Cascade complexes are named according to the alteration of the WT spacer length. Target DNA bearing CC PAM and protospacer, matching to all spacers, is depicted below. Schematic representation of WT, −12 and +12 Cascade complexes is shown on the right. **b** Phage and **c** plasmid interference mediated by altered-length Cascade complexes. Phage interference is represented as ratios of PFU of M13-NT (non-target) and M13-SP-CC (target) phages in the context of Cascade complexes only. Plasmid interference is depicted as transformation efficiencies (CFU/μg of DNA) of pNT (non-target) or pSP-CC (target) plasmid DNA in the background of the complete Aa-CRISPR-Cas system. **d** Composition of purified Cascade complexes. SDS-PAGE of purified protein preparations is presented on the top. Denaturing polyacrylamide gel (on the bottom) shows extracted crRNA from the altered Cascade complexes. **e** Binding of altered-length Cascade complexes to DNA. Binding of Cascade variant to non-target (NT; on the top) or target (SP-CC; on the bottom) DNA was monitored by EMSA. **f** Binding affinities of altered-length Cascade complexes to target and non-target DNA. Dissociation constant *K*_d_ values were obtained by EMSA. **g** Triggering of target DNA degradation by altered-length Cascade complexes. Cleavage of the linearized plasmid (pSP-CC) was monitored in the presence of Cas2/3 and Cascade variant. Error bars in the **b**, **c,** and **f** represent standard deviations in at least three separate experiments (individual data values are provided in Additional file 14)

Length of spacers in CRISPR regions is predetermined by adaptation machinery, which inserts spacers in a ruler-like manner [51]; therefore, spacers of defined length dominate in the locus. Sometimes, the ruler fails or CRISPR locus rearranges; thus, spacers of different length emerge in the array [41, 52, 53]. The most common spacer length in Aa-CRISPR region is 32 bp (WT); however, spacers of different length could be found as well (Additional file 1: Table S1). Six Cas7f1 subunits of the type I-F1 Cascade surround the WT spacer of the crRNA. The first Cas7f1 subunit interacts with 4 nt of 5′-handle as well as 2 nt of the spacer, while other five Cas7f1 subunits bind spacer sequence in 6 nt increments [43]. Based on structural data, the number of Cas7f1 subunits (*N*) is proportional to the spacer length (*L*) according to a relationship *L = 6·N − 4*; e.g. spacer of 20 or 44 should potentially interact with 4 or 8 subunits of Cas7f1, respectively (Fig. 3a).

It is not known whether longer or shorter spacers permit assembly of functional type I-F1 Cascade complex that confers DNA interference. To address this, we first constructed expression vectors for CRISPR loci where the 3′-end of the WT spacer was truncated or extended by the increments of 6 bp, corresponding to the interaction site of Cas7f1. We truncated the spacer up to 2 bp, which potentially could be assembled into minimal Cascade complex with one copy of each protein subunit. On the contrary, the longest spacer examined was of 176-bp length and presumably could contain Cas7f1 backbone of 30 subunits. The engineered spacers could be in principle incorporated into Cascade complexes, which throughout the text are named according to the alteration of spacer length, i.e. −30, −24, −18, −12, and −6 for the complexes with truncated spacer (truncated complexes) and +6, +12, +18, +24, +30, +54, +84, and +144 for the complexes with extended spacer (extended complexes) (Fig. 3a).

Then functionality of altered Cascade complexes was assayed in vivo using DNA targets M13-SP-CC and pSP-CC containing CC PAM and a protospacer sequence, which is complementary to all spacer variants (Fig. 3a). Initially, we tested a level of M13-SP-CC phage infection in the context of the Cascade complexes, which can be extrapolated to Cascade binding efficiency in vivo. Extended Cascade complexes impeded phage infection with similar efficiency as the WT Cascade complex. On the contrary, suppression of phage infection decreases gradually according to shortening of spacer length. When the spacer is truncated by 18 nt or more, the level of phage infection is similar to the complex containing non-targeting spacer (Fig. 3b). Next, we analysed the interference of pUC-SP-CC plasmid in the context of the complete Aa-CRISPR-Cas system that reflects DNA degradation efficiency in vivo. Transformation efficiency of DNA target in the presence of extended complexes is similar to the WT, while even −6 Cascade does not stimulate interference of DNA target (Fig. 3c). To conclude, Cascade complexes with extended spacers bind and stimulate DNA interference at a similar extent as WT Cascade in vivo. On the other hand, the truncation of spacer decreases the binding efficiency of Cascade and intercepts DNA interference in vivo.

Ultimately, we performed pull-down experiments to examine if all altered-length spacers can be incorporated into stable Cascade complexes. We did not manage to purify −30 and −24 complexes; thus, the length of these spacers is probably too short to maintain assembly of stable Cascade complex. All other complexes starting from −18 were pulled down successfully. These complexes contain all protein subunits of the Cascade and crRNA as judged from nucleic acid extraction. Lengthening of spacer correlates with the increasing amount of Cas7f1 and with the extension of crRNA (Fig. 3d). Although increasing amounts of degradation products are seen below the dominant band in the samples of extended crRNAs, the degradation bands comprise only a small fraction in the total nucleic acid extracts (e.g. the band of highest molecular weight corresponds to ~ 97% of total intensity in the lane of +144 crRNA, while all additional bands comprise only about 3%). Thus, Cascade complexes with full-length crRNA should dominate in the purified sample.

### Spacer length controls DNA degradation stage

We examined the purified Cascade complexes in vitro to validate in vivo results. First, we tested if the Cascade complexes can discriminate and bind DNA target, which carried CC PAM and protospacer with complete complementarity for all the spacers (Fig. 3e). Weak binding to DNA target can be seen for −18 Cascade when compared with the binding to the non-target DNA. Complexes from −12 up to +144 interact with the DNA target, while the non-target DNA is not bound. Furthermore, DNA shift in the gel is proportional to the length of the spacer in the Cascade complex, i.e. the longer the Cascade complex, the slower the band corresponding to the Cascade-bound DNA is migrating.

To evaluate the binding quantitatively, we measured dissociation constants for representative Cascade complexes: −18, −12, −6, WT, +30, and +144. All complexes bind the non-target DNA with a comparable affinity (Fig. 3f). Dissociation constants of extended complexes for target DNA are similar to WT and are about fourfold lower than the constants for the non-target DNA. On the contrary, the increase of *K*_d_ values for truncated Cascades are proportional to the spacer length decrease.

Next, using footprint assay, we monitored whether Cascade binding to target DNA leads to an R-loop formation. The non-complementary DNA strand of the protospacer is displaced as an ssDNA in the R-loop; thus, it becomes more susceptible to KMnO_4_ treatment. Footprint analysis revealed that all complexes form R-loops upon binding to the DNA target and the length of the R-loop depends on the spacer length (Additional file 6: Figure S5).

Then, we assayed whether different Cascade complexes can trigger Cas2/3 to degrade DNA target. Linearized plasmid DNA was pre-bound with Cascade complexes and plasmid degradation was initiated by addition of Cas2/3 protein. The extended Cascades, as well as WT Cascade complex, stimulate DNA degradation. However, target DNA bound by the truncated Cascade complexes is not hydrolysed (Fig. 3g).

Finally, we assayed cleavage positions of Cas2/3 on the labelled DNA fragment bound by the −18, −12, −6, WT, +30, and +144 Cascade complexes. Following plasmid hydrolysis data, Cas2/3 does not cleave DNA targeted by the −18, −12, and −6 complexes neither in the absence nor in the presence of ATP (Additional file 7: Figure S6 and Additional file 8: Figure S7). On the other hand, WT or longer Cascade complexes trigger Cas2/3 to hydrolyse the targeted DNA. In the absence of ATP, hydrolysis products are mapped within the displaced DNA strand at around 25th position from the PAM (Additional file 7: Figure S6), while degradation pattern extends upstream from these positions in the displaced strand when Cas2/3 is stimulated by ATP hydrolysis (Additional file 8: Figure S7). The Cascade-targeted strand is cleaved only in the presence of ATP at the PAM-proximal region (approximately 7th–17th positions from the PAM within the protospacer region for the WT and extended complexes and also approximately 24th–32nd positions for the extended complexes; Additional file 8: Figure S7).

### PAM-distal end of the 32 bp R-loop triggers DNA degradation

**Fig. 4:**
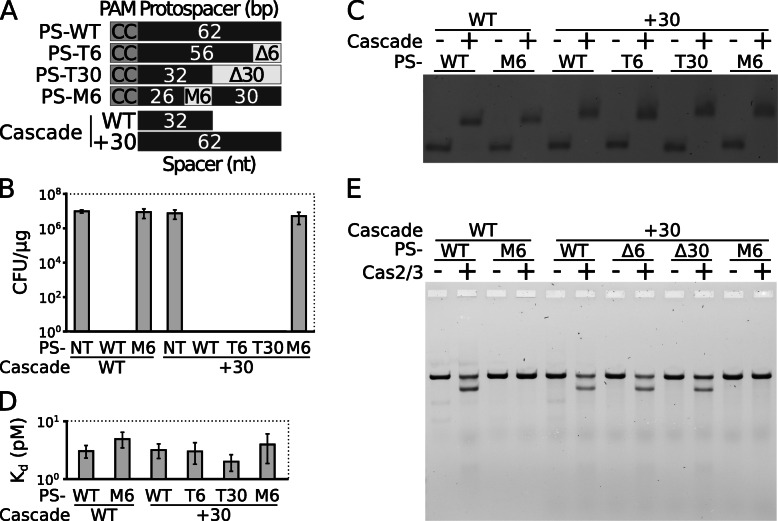
Influence of protospacer mutations at the PAM-distal end on DNA interference. **a** Constructs of altered protospacer sequences. The CC PAM-distal end of protospacer (PS-CC), which has complete complementarity for 32 and 62 nt spacers, was truncated by 6 (PS-T6) or 30 bp (PS-T30) or a mismatch of 6 bp was introduced between the 27th and 32nd positions of the protospacer (PS-M6). **b** Interference for plasmids with altered protospacer sequences. Plasmids containing either WT or altered target sequence (as shown in **a**) as well as plasmid without target sequence (PS-NT) were introduced into *E. coli* cells carrying Cas1-Cas2/3 and WT or +30 Cascade; then, transformation efficiency was estimated (CFU per μg of plasmid DNA). **c** WT and +30 Cascade binding to DNA with altered protospacer sequences. Binding of WT and +30 Cascade to respective DNA was examined by the EMSA. **d** Dissociation constants for all substrates. The constants were determined using EMSA. **e** Dependency of DNA hydrolysis on R-loop length. Cleavage of respective linearized plasmid was monitored in the presence of Cas2/3 and WT or +30 Cascade. Error bars in **b** and **d** represent standard deviations for at least three separate experiments (individual data values are provided in Additional file 14)

Cascade complexes with truncated spacers form R-loops upon target binding; however, they do not trigger Cas2/3-mediated DNA degradation. We were wondering whether truncation of CC PAM-distal end of the protospacer leads to similar effects mediating WT and extended Cascade complexes. Therefore, the PAM-distal end of the 62-bp protospacer (PS-WT) was truncated by 6 and 30 bp (PS-T6 and PS-T30, respectively), which should form R-loops of 56 or 32 bp lengths upon +30 Cascade binding, respectively. Besides, the sequence between 27 and 32 bp positions of the protospacer was replaced with 6 bp mismatching sequence (PS-M6). WT and +30 Cascade should form the R-loop of 26 bp within the PS-M6 substrate, whereas the +30 Cascade can potentially extend the R-loop up to 62 bp by overriding the mismatching region (Fig. 4a).

To examine the consequence of protospacer length change for DNA interference, we assayed transformation efficiencies of plasmids bearing mutated protospacers in the context of Aa-CRISPR-Cas system. Similarly to −6 Cascade complex (Fig 3c), interference mediated by WT Cascade complex is inhibited when 32-bp protospacer is truncated by 6 bp (PS-M6; Fig. 4b). On the contrary, the truncation of 62-bp protospacer by 6 or even 30 bp maintains DNA interference triggered by +30 Cascade; however, +30 Cascade-mediated interference is impeded by PS-M6.

We examined whether inhibition of DNA interference can be associated with impaired Cascade binding. All mutated protospacers are bound specifically by WT and +30 Cascade complexes (Fig. 4c). Furthermore, dissociation constants for these substrates are similar to PS-WT (Fig. 4d). We performed footprint assay to test whether specific Cascade binding leads to the R-loop formation of the expected length. As anticipated, WT and +30 Cascade form R-loop of 26 bp upon binding to the PS-M6, while 56 and 32 nt of the +30 Cascade spacer basepair with the PS-T6 and PS-T30 protospacer, respectively (Additional file 9: Figure S8).

Finally, we analysed if Cascade binding to these substrates can trigger Cas2/3 nuclease activity. In correspondence with in vivo results, WT or +30 Cascade complexes do not trigger hydrolysis of the PS-M6 substrate, while truncation of 62-bp protospacer by 6 or 30 bp does not influence +30 Cascade-mediated DNA degradation (Fig. 4e). Furthermore, the cleavage pattern of Cas2/3 is similar for these substrates (Additional file 10: Figure S9). To conclude, Cas2/3-mediated DNA degradation is impeded by either truncation of the WT spacer or the mismatch at the PAM-distal end of the 32-bp length protospacer what leads to shortening of the 32-bp length R-loop.

## Discussion

The widely spread and one of the most diverse type I CRISPR-Cas systems gain a huge interest as powerful molecular tools [18–21, 54]. However, knowledge of the type I operation is based on extensive biochemical and structural studies of the subtype E, while the research is sparse for other subtypes. In this study, using in vivo and in vitro assays, we examine the interference stage in the type I-F1 CRISPR-Cas system from *A. actinomycetemcomitans* D7S-1 bacteria and compare it with the type I-E interference.

Type I systems identify DNA as a non-self when PAM sequence is present next to protospacer. Aligning spacer-matching phage genome sequences, we found a conservative CC dinucleotide sequence immediately upstream of the protospacer, which serves as the PAM in Aa-CRISPR-Cas system. Identical PAM sequences are recognised by other complexes of type I-F1 (Pae- and Pat-Cascade) and even I-F2 [41, 50, 55]. We examined all dinucleotide variants in −2, −1 position upstream the protospacer to see full spectra of PAM sequences. We found that only a single C at the −1 position or CT sequence (−2, −1 position) is required for foreign DNA discrimination (Fig. 1). Similar PAM promiscuity was demonstrated previously for the type I-E Cascade where multiple PAMs can be tolerated with the most critical base pair at −1 position [34, 56]. Recently, the structures of Pae-Cascade bound to a DNA target were solved, which reveal that type I-F1 Cascade, likewise, the type I-E, recognises PAM sequence from the minor DNA groove griping dsDNA between Cas7f1 and the large subunit Cas8f1 [42, 57]. The Cas8f1 makes direct contacts with both G nucleotides of the PAM (complementary to the CC); however, the more extensive interactions with G(−1) explain the dominance of C/G pair in the −1 position. Differently from type I-E and I-F1, the type I-F2 Cascade recognises PAM from the major DNA groove. This complex lacks the large subunit (Cas8), and PAM is recognised by an alpha-helical domain, which is fused to Cas5f2 protein [58].

PAM sequence is the initiation site for the formation of the R-loop [50] where spacer basepairs with the complementary DNA strand of protospacer displacing the non-complementary strand as ssDNA. As expected, Aa-Cascade binds specifically to DNA target forming the R-loop structure, which triggers the ssDNA nuclease/helicase Cas2/3 to degrade the target (Fig. 2). Cascade-triggered DNA target degradation was already demonstrated in the type I-F1 Pae-CRISPR-Cas system [39]. Here we provide a more detailed molecular mechanism of DNA interference. We show that Aa-Cascade positions the Cas2/3 to cleave within the displaced strand at 22nd–25th nt from the PAM where ATP-fuelled unidirectional degradation is initiated (Fig. 2, Additional file 4: Figure S3 and Additional file 5: Figure S4). The degradation extends upstream from this site on the non-target strand, while hydrolysis of the target strand is terminated within the protospacer region. Although this overall Cas2/3 degradation profile resembles Cas3 of the type I-E [34], cleavage of Cas2/3 and Cas3 is initiated at different positions. Upon R-loop formation, Cas11 subunits of the type I-E Cascade lock the R-loop and bulge PAM-proximal end of the non-target strand of the protospacer [32] where Cas3 docks and introduces cuts within 6th–15th nt positions counting from the PAM [33, 34]. In the type I-F1, cleavage positions of the Cas2/3 are shifted to the PAM-distal end of the non-target strand (22nd - 25th positions) and overlap with the accessible region (18th–25th positions) of P1 endonuclease (Additional file 3: Figure S2), which presumably corresponds to the bulge in the protospacer’s non-target strand. To sum up, the type I-F1 and I-E Cascade complexes dock the nuclease/helicase at different sites, where DNA degradation is initiated (Additional file 11: Figure S10A).

Cascade complexes in the type I-E and type I-F2 can incorporate spacers, which length differ from the original spacer length found in WT complex [52, 55, 59, 60]. Recently, RNA length was shown to govern the oligomeric state of isolated *Zymomonas mobilis* Cas7f1 [61]. We wanted to determine the range of spacer lengths, which can be incorporated into stable type I-F1 Cascade complex. We show that the lower limit of spacer length is about 14 nt, which should be embedded in the Cascade backbone of three Cas7f1 subunits. The largest complex that we purified contains spacer of 176-nt length that presumably is assembled into the backbone of 30 Cas7f1 subunits (Fig. 3a and d). The assembly of such extended complexes most likely depends on the production of Cas7f1 subunits that polymerise on spacer region of crRNA. If cellular resources of Cas7f1 are low, a fraction of long spacers might be incompletely covered and could be cleaved by endogenous RNases, which would lead to the emergence of two Cascade subspecies: (i) 3′- and (ii) 5′-subcomplex. The 3′-subcomplex contains 3′-end of crRNA and only Cas7f1 and Cas6f proteins; thus, it is incapable to initiate R-loop formation. On the other hand, the 5′-subcomplex composed of 5′-part of crRNA as well as Cas8f1, Cas5f1, and Cas7f1 proteins can in principle recognise and interact with DNA target; therefore, it might assist the extended Cascade complex to interfere with DNA target in vivo.

All purified complexes bound to target DNA specifically, although binding affinities for target DNA decreased proportionally to spacer length shortening in the truncated complexes. On the other hand, extended complexes bind to target DNA with similar affinity to WT Cascade, which is in agreement with in vivo results (Fig. 3). Upon target binding, all Cascade complexes form R-loops, which length correlate with the spacer length (Additional file 6: Figure S5). Further, we demonstrate that the extended complexes trigger Cas2/3 to degrade DNA target, while R-loops formed by the truncated Cascades do not induce Cas2/3 cleavage (Additional file 7: Figure S6 and Additional file 8 Figure S7). Moreover, cleavage profile of the Cas2/3 remains similar despite R-loop extension implying that the docking surface for the Cas2/3 is localised at the same site within both WT and extended Cascade complexes most likely at the interface of Cas8f1, Cas5f1, or both subunits. Different from the type I-F1, the type I-E Cascade complexes bearing truncated spacers by 6 or 12 bp maintain the Cas3-mediated target degradation, although DNA hydrolysis rate is markedly reduced [52, 60]. The docking site for Cas3 (type I-E) is localised at the PAM-proximal end of the WT R-loop; thus, truncations of the R-loop presumably do no influence Cas3 loading, while the Cas2/3 (type I-F1) is loaded at the PAM-distal end of the WT R-loop where truncation of even 6 bp abolishes the Cas2/3-mediated DNA degradation (Additional file 11: Figure S10B and S10C).

We modified protospacer sequence, thereby disrupting base-pairing with either extended +30 or WT Cascade at the PAM-distal end (Fig. 4). We show that the Cascade complexes bind truncated DNA substrates with similar affinity to WT protospacer forming R-loops of the expected length (Additional file 9: Figure S8). The extended Cascade triggers degradation of protospacer truncated to WT length of 32 bp; however, when a mismatch of 6 bp is introduced within the protospacer (27th–32nd bp from the PAM), both WT and the extended Cascades do not stimulate the Cas2/3 nuclease (Fig. 4 and Additional file 10: Figure S9). On the contrast, the extended type I-E Cascade can overcome analogous mismatches within the protospacer and stimulate DNA hydrolysis to a similar extent of protospacer with complete complementarity [60]. The presence of small subunits Cas11 in the type I-E Cascade complex might be responsible for facilitated formation and stabilisation of such R-loops (Additional file 11: Figure S10D and S10E).

**Fig. 5:**
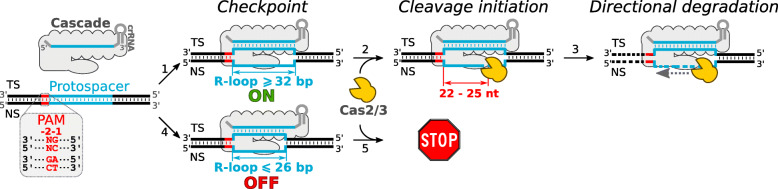
DNA interference in the type I-F1 CRISPR-Cas system. Ribonucleoprotein Cascade complex scans DNA for a complementary protospacer, which is adjacent to the promiscuous PAM sequence. Upon DNA target recognition, Cascade complex forms an R-loop (1 and 4) where the spacer of the crRNA basepairs with the target strand (TS) of the protospacer displacing the non-target strand (NS) as an ssDNA. In a checkpoint step of DNA interference, Cascade “measures” the length of the R-loop. When R-loop reaches WT length of 32 bp or is extended above this length by Cascade complex containing longer spacer (1), DNA interference is turned on. Then, the helicase/nuclease Cas2/3 docks to the NS of the R-loop and initiates cleavage (2) at the PAM-distal end of the R-loop (22nd-25th nt from the PAM) followed by unidirectional DNA degradation upstream from the protospacer, while leaving the downstream DNA intact (3). On the contrast, truncated Cascade or mismatches at the PAM-distal end of the protospacer that mediate the formation of 26 bp length or shorter R-loops (4) prevent cleavage initiation (5); thus, DNA interference is turned off

These results reveal an on/off switch for initiation of the type I-F1 DNA interference, which is tightly controlled by the R-loop length. Cascade initiates DNA degradation when R-loop of WT length (32 bp) is formed, while shorter R-loops keep DNA interference at the turned-off state (Fig. 5). Our data are supported by the recently solved structure of Pae-Cascade bound to the DNA target where a conformational change of the C-terminal domain of Cas8f1 was captured [57]. Upon full-length R-loop formation, this domain is reoriented to the PAM-distal end of the R-loop and probably serves as a hub for Cas2/3 loading on the displaced DNA strand of the R-loop, which is subsequently degraded. When the R-loop of 32 bp length is truncated, the reorientation of the C-terminal domain is most likely blocked by steric hindrance, thereby intercepting Cas2/3 binding or activation. On the other hand, the spacer-protospacer complementarity of 32 bp is enough for the domain reorientation and subsequent Cas2/3 triggering despite the final length of the R-loop.

## Conclusions

We provide a comprehensive in vivo and biochemical analysis of DNA interference mechanism in the type I-F1 CRISPR-Cas system and disclose differences from the type I-E DNA interference (Additional file 11: Figure S10). We demonstrate the flexibility of type I-F1 Cascade complex to incorporate crRNA of different than WT length. Our results reveal a checkpoint for the type I-F1 DNA interference, which is controlled by base-pairing at the PAM-distal end of the WT R-loop. When R-loop of 32-bp (WT) length or longer is formed, Cascade turns on DNA interference by loading Cas2/3 nuclease/helicase at the PAM-distal end of the protospacer’s non-target strand where ATP-fuelled unidirectional degradation of DNA target is initiated. Otherwise, R-loops of 26-bp length or shorter keep DNA interference in the turned-off state (Fig. 5).

## Methods

### Cloning, expression, and purification of proteins

Genes encoding Cascade, Cas1, and Cas2/3 proteins were amplified using *A. actinomytetemcomitans* D7S1 genomic DNA, while CRISPR locus containing a single repeat-spacer-repeat unit was assembled from oligonucleotides. All components of the Aa-CRISPR-Cas system were cloned under the inducible T7 promoter. DNA targets were cloned into pUC19 vector or M13mp18 bacteriophage genome. All DNA constructs are listed in the Additional file 12: Table S2 [34, 62], while oligonucleotides used for cloning are provided in the Additional file 13: Table S3.

The Cas2/3 protein and Cascade complexes were expressed in *Escherichia coli* BL21-AI, which was either transformed with pCas2/3-H encoding Cas2/3 fused with His_6_-tag at C-terminus or co-transformed with pCd-H plasmid encoding Cas8f1, Cas5f1, Cas7f1, and Cas6f protein with N-terminal His-tag and plasmid with CRISPR region containing spacer of certain length (Additional file 12: Table S2), respectively. Cells were grown in LB broth (BD) supplemented with respective antibiotic (50 μg/ml ampicillin or 25 μg/ml streptomycin and 17 μg/ml chloramphenicol) at 37 °C to OD600nm of ~ 0.5, and expression was induced with 1 mM IPTG for 3–4 h. Harvested cells were disrupted by sonication; cell debris removed by centrifugation. The supernatant with the Cas2/3 protein was loaded on the Ni^2+^-charged HiTrap column (GE Healthcare) followed by Superdex 200 (HiLoad 16/60; GE Healthcare), while Cascade was purified by subsequent Ni^2+^-charged HiTrap column (GE Healthcare), Superdex 200 (HiLoad 16/60; GE Healthcare), and heparin (GE Healthcare) chromatography steps. The Cas2/3 was stored at − 20 °C in a buffer containing 20 mM HEPES (pH 7.3), 500 mM NaCl, 50% (v/v) glycerol, and 2 mM DTT, while buffer for storage of Cascade complexes was 20 mM Tris–HCl (pH 8.0), 500 mM NaCl and 50% (v/v) glycerol, and 2 mM DTT. The proteins were further analysed by 12% SDS-PAGE. The concentration for Cas2/3 was measured by UV (280 nm) absorbance, while concentration for Cascade complexes was estimated by Pierce™ 660 nm protein assay using BSA as a reference. Conversion to the molar concentration of Cascade complexes was performed assuming the stoichiometry Cas8f1_1_:Cas5f1_1_:Cas7f1_N_:Cas6f_1_:crRNA_1_ where the number of Cas7 subunits (*N*) corresponds to spacer length (*L*) in such relationship: *L = 6·N − 4*.

### Plasmid interference assays

Cas protein-encoding plasmid (for determination of components of interference pCd-Cas1–2/3, pCd, pCas1–2/3, or empty plasmid was used; for PAM promiscuity—pCd and pCOLA-Cas2/3; for spacer length and protospacer mutations—pCd-Cas1–2/3) together with a plasmid containing CRISPR region (for determination of components of interference and PAM promiscuity pCR (WT) with a spacer of 32 bp was used; for spacer length—spacer sequences ranged in 6-bp increments from 2 to 176 bp (Additional file 12: Table S2); for protospacer mutations—pCR (WT and +30)) was inserted into *E. coli* BL21-AI. The chemically competent cells were transformed with plasmids bearing target sequence (pSP-CC or its derivatives; Additional file 12: Table S2) and plasmid without target sequence (pNT). Tenfold serial dilutions of the transformed cells were plated on LB-agar medium containing 0.1 mM IPTG, 0.2% (w/v) l-arabinose, chloramphenicol (17 μg/ml), streptomycin (25 μg/ml), and carbenicillin (25 μg/ml) (15 μg/ml of kanamycin was also added in the PAM promiscuity experiment). The efficiency of plasmid interference was estimated from transformation efficiencies expressed as CFU per μg of transformed plasmid DNA (see Additional file 14 for individual data values).

### Phage interference assays

Cas protein-encoding plasmid (for determination of components of interference pCd-Cas1–2/3, pCd, pCas1–2/3, or empty plasmid was used; for spacer length—pCd) together with a plasmid containing CRISPR region (for determination of components of interference pCR (WT) plasmid was used; for spacer length—spacer sequences ranged in 6-bp increments from 2 to 176 bp (Additional file 12: Table S2)) was inserted into *E. coli* Nova Blue. The overnight cultures were mixed with soft LB (0.75%) agar, which was spread on precast LB (1.5%) agar medium containing chloramphenicol (17 μg/ml) and streptomycin (25 μg/ml). Tenfold serial dilutions (10^−1^–10^−8^) of M13-SP-CC (with target sequence) and M13-NT (without target sequence) in LB medium were placed as 10 μl drops on soft agar. The efficiency of phage interference was estimated as the ratio of M13-NT and M13-SP-CC PFU in a background of respective Aa-CRISPR-Cas plasmids (see Additional file 14 for individual data values).

### Extraction of crRNA

Nucleic acids co-purified with Cascade were isolated by phenol to chloroform to isoamyl alcohol (PCI) (25:24:1, v/v/v) extraction. Nucleic acids were separated by electrophoresis on a denaturing 15% polyacrylamide gel and visualised with SYBR Gold (Invitrogen).

### Cas2/3 nuclease assay

Nuclease activity of Cas2/3 was assayed at 37 °C for 30 min in a nuclease (N1) buffer (20 mM HEPES (pH 7.3 at 25 °C), 100 mM NaCl, 5% (v/v) glycerol, 0.1 mg/ml BSA, 5 mM CoCl_2_) supplemented with 5 nM single-stranded (ss) circular M13mp18 DNA or double-stranded (ds) supercoiled pSP-CC DNA. Reactions were initiated by adding 10 nM Cas2/3 and stopped by mixing with 2x Stop solution (75 mM EDTA, 50% (v/v) glycerol, 0.25% (w/v) SDS, OrangeG).

Influence of different divalent metal ions for Cas2/3 nuclease activity was examined at 37 °C for 30 min in the N1 buffer supplemented with 5 nM ssM13mp18 and 0.5 mM or 5 mM divalent metal ions: Mg^2+^ (MgCl_2_), Ca^2+^ (CaCl_2_), Mn^2+^ (MnCl_2_), Co^2+^ (CoCl_2_), Ni^2+^ (NiCl_2_), or Zn^2+^ (ZnCl_2_). The reactions were initiated by introducing 10 nM Cas2/3 and terminated by mixing with 2x Stop solution. Products of the reactions were fractionated by 1% (w/v) agarose gel electrophoresis and visualised with ethidium bromide or SYBR Gold.

### Cas2/3 ATPase assay

ATPase activity of the Cas2/3 was assayed at 37 °C in an ATPase (A) buffer (10 mM Tris–HCl (pH 8.0 at 25 °C), 75 mM NaCl, 7% (v/v) glycerol, 0.1 mg/ml BSA, 2 mM MgCl_2_, 2 mM ATP and 1 nM of [α^32^P] ATP (Hartmann Analytic)) in the presence of 5 nM double-stranded pSP-CC plasmid or single-stranded M13mp18 or in the absence of DNA. Reactions were initiated by adding Cas2/3 to the reaction mixture and terminated at different time points (0–64 min) by the addition of EDTA to 25 mM final concentration. Reaction products were fractionated on thin-layer chromatography (TLC) PEI Cellulose F plates (Merck) in 0.325 M sodium phosphate buffer (pH 3.5 at 23 °C) and visualised using FLA-5100 phosphorimager (Fujifilm). The ATP depletion was measured as a function of time and fitted according to a first-order kinetical model *[ATP]*_*t*_ *= [ATP]*_*o*_*e*^*−kt*^. The reaction rate constants were estimated from at least three independent measurements (see Additional file 14 for individual data values).

Influence of different divalent metal ions for Cas2/3 ATPase activity was examined at 37 °C for 20 min in the A buffer containing 5 nM ssM13mp18 and 5 mM of divalent metal ions: Mg^2+^ (MgCl_2_), Ca^2+^ (CaCl_2_), Mn^2+^ (MnCl_2_), Co^2+^ (CoCl_2_), Ni^2+^ (NiCl_2_), or Zn^2+^ (ZnCl_2_).

### Electrophoretic mobility shift assays (EMSA)

#### EMSA in agarose gel

To monitor Cascade-DNA interactions, 30 nM of Cascade complexes were mixed with 10 nM SP-CC-1, SP-AA-1, or NT-1 DNA fragments (Additional file 13: Table S3) in a Binding (B) buffer (40 mM Tris-HCl (pH 8.3 at 25 °C), 20 mM acetic acid, 1 mM EDTA, 125 mM NaCl, 12% (v/v) glycerol, 0.1 mg/ml BSA) and incubated at 37 °C for 5 min. The samples were separated on 1% (w/v) agarose gel prepared in 1x TAE buffer (Thermo Fisher) and visualised by ethidium bromide staining.

#### EMSA in polyacrylamide gel

To estimate binding affinity for Cascade complexes, 0.01 nM of ^32^P-5′-end-labelled SP1-CC-2 (target) and SP3-AA-2 (non-target) DNA fragments in the B buffer were incubated at room temperature for 10 min with increasing concentration of Cascade complexes. The samples were separated on 8% (w/v) polyacrylamide gel prepared in 1x TAE buffer (Thermo Fisher) for 2 h 30 min at 5 V/cm and visualised using an FLA-5100 phosphorimager (Fujifilm). *K*_d_ values were calculated as previously described by Tamulaitis et al. [63] from three independent experiments (see Additional file 14 for individual data values).

### R-loop footprinting experiments

#### P1 nuclease footprinting

Oligoduplex SP-CC was ^33^P-5′-end-labelled at either the target or non-target strand, while only the non-target strand of the SP-AC, SP-TC, and SP-GC oligoduplexes was labelled (Additional file 13: Table S3). Then, the labelled oligoduplexes were incubated with the WT Cascade and treated with P1 nuclease as described previously [34].

#### KMnO_4_ footprinting

DNA fragment SP-CC-3 was ^32^P-5′-end-labelled at the non-target strand (Additional file 13: Table S3) and then at 10 nM concentration was incubated with or without Cascade complex (400 nM of the −18 and −12 Cascade complexes was used, while −6 to +144 Cascade complexes were incubated at 100 nM) at 37 °C for 5 min in the B buffer following KMnO_4_ (Sigma) addition to the final concentration of 2 mM. The reactions were terminated with 0.5 M β-mercaptoethanol and 0.75 M sodium acetate (pH 7.0 at 25 °C) buffer after incubation at 37 °C for 20 or 60 s. The stopped reactions were dried by vacuum centrifugation at 60 °C for 60 min, followed by addition of 1 M piperidine and 30-min incubation at 90 °C. The nucleic acids were precipitated by sodium acetate/isopropanol and solubilised in 2x loading dye (95% (v/v) formamide, 0.5 mM EDTA, 0.025% bromophenol). The products were separated on a denaturing 20% polyacrylamide gel and visualised by autoradiography. The ^32^P-5′-end-labelled M-32, M-62, M-116, and M-176 DNA duplexes together with products of dideoxy sequencing reactions (“Cycler Reader DNA Sequencing kit” (Fermentas)) using ^32^P-5′-end-labelled TS931 oligonucleotide were utilised as size markers for the non-target strand of the SP-CC-3 (Additional file 13: Table S3).

### Cas2/3 cleavage assay in the presence of Cascade complexes

#### Non-labelled DNA targets

Supercoiled pSP-CC, pSP-AA, and pNT plasmids, as well as pSP-CC plasmid linearized with either SacI, SspI, Eam1105I, CaiI, or LguI (Thermo Fisher) restriction endonucleases, were used as substrates in the DNA cleavage assay. The DNA substrate at 5 nM was pre-incubated with 30 nM Cascade complex at 37 °C for 5 min in a Nuclease (N2) buffer (20 mM HEPES (pH 7.3 at 25 °C), 100 mM NaCl, 7% (v/v) glycerol, 0.1 mg/ml BSA, 1.5 mM CoCl_2_, 2 mM ATP, 25 mM phosphocreatine (Sigma Aldrich), 5 mU/μL Creatine Phosphokinase from rabbit muscle (Sigma Aldrich)); then, 1000 nM (or indicated concentration) Cas2/3 was added to initiate nuclease reactions. After incubation at 37 °C for 1 h, the reactions were terminated by mixing with 2x Stop solution followed by heating at 75 °C for 15 min. The samples were separated on 1% (w/v) agarose gel and visualised by ethidium bromide staining.

#### Labelled DNA targets

SP-CC oligoduplex and SP-CC-3 DNA fragment ^32^P-5′-end-labelled on either target or non-target strand were used to map cleavage positions of the Cas2/3. Cascade complex at 5 or 60 nM was pre-incubated with 1 nM SP-CC or 10 nM SP-CC-3, respectively, at 37 °C for 5 min in the N2 buffer; then, 500 nM (or indicated amount) Cas2/3 was added to initiate nuclease reactions. After incubation at 37 °C for 1 h, the reactions were terminated by mixing with 2x Stop solution. The nucleic acids were precipitated by sodium acetate/isopropanol/glycogen and solubilised in 2x loading dye. The products of SP-CC and SP-CC-3 were separated on a denaturing 20 and 15% polyacrylamide gel, respectively, and visualised by autoradiography. Products of dideoxy sequencing reactions (“Cycler Reader DNA Sequencing kit” (Fermentas)) of ^32^P-5′-end-labelled TS153 or TS931 and TS166 or TS930 oligonucleotides were used as size markers for the non-target strand and target strand of the SP-CC or SP-CC-3, respectively (Additional file 13: Table S3).

## Supplementary information

**Additional file 1: Table S1.** Spacer sequences in the CRISPR2 region of *Aggregatibacter actinomycetemcomitans* D7S-1.

**Additional file 2: Figure S1.** Cas2/3 nuclease and ATPase activity dependence on divalent metal ions. (**A**) Cas2/3-catalysed hydrolysis of ssDNA in the presence of different divalent metals. (**B**) Cas2/3-catalysed hydrolysis of ATP in the presence of different divalent metals.

**Additional file 3: Figure S2.** R-loop formed by WT Cascade. (**A**) Denaturing polyacrylamide gel of oligoduplex footprints. (**B**) R-loop within SP-CC oligoduplex sequence.

**Additional file 4: Figure S3.** Cas2/3-mediated DNA degradation. (**A**) Preparation of linearized plasmid substrates. (**B**) Cas2/3 degradation of Cascade-bound linearized DNA targets.

**Additional file 5: Figure S4.** Cas2/3 cleavage profile of Cascade-bound target dsDNA. Denaturing polyacrylamide gels of Cas2/3-cleaved oligoduplex labelled on either the non-target (**A**) or target (**B**) DNA strand. (**C**) Cleavage products mapped within the SP-CC oligoduplex sequence.

**Additional file 6: Figure S5.** R-loops formed by Cascade complexes containing truncated or extended spacers. Denaturing polyacrylamide gels of DNA duplex footprints after treatment with KMnO_4_ for a 60 s (**A**) or 20 s (**B**). (**C**) Cleavage pattern within the the non-target strand sequence.

**Additional file 7: Figure S6.** Cas2/3 cleavage of target DNA bound to truncated and extended Cascade complexes in the absence of ATP. Denaturing polyacrylamide gels of Cas2/3-cleaved DNA target labelled on either the non-target (**A**) or target (**B**) DNA strand. (**C**) Cleavage pattern within the DNA target.

**Additional file 8: Figure S7.** Cas2/3 cleavage of target DNA bound to truncated and extended Cascade complexes in the presence of ATP. Denaturing polyacrylamide gels of Cas2/3-cleaved DNA target labelled on either the non-target (**A**) or target (**B**) DNA strand. (**C**) Cleavage pattern within the DNA target.

**Additional file 9: Figure S8.** R-loops formed by WT and extended Cascade upon interaction with protospacers of different length. (**A**) Denaturing polyacrylamide gel of DNA duplex footprints. (**B**) Cleavage pattern within the non-target strand sequences.

**Additional file 10: Figure S9.** Cas2/3 cleavage within protospacers of different length. Denaturing polyacrylamide gels of Cas2/3-cleaved DNA targets in the absence (**A**) or presence (**B**) of ATP. (**C**) Cleavage pattern within the non-target strand sequences.

**Additional file 11: Figure S10.** Comparison of DNA interference stages in the type I-F1 and I-E CRISPR-Cas systems. (**A**) DNA interference mediated by the R-loop of WT length. (**B**) DNA interference mediated by the extended Cascade. (**C**) DNA interference mediated by the truncated Cascade. (**D**) Interference of DNA containing the truncated protospacer. (**E**) Interference of DNA containing a mismatch within the protospacer.

**Additional file 12: Table S2.** Expression vectors and plasmid substrates.

**Additional file 13: Table S3.** Oligonucleotides.

**Additional file 14.** Individual data values. Values are provided for: Fig. 1: pannels D, E; Fig. 2: pannel D; Fig. 3: pannels B, C; F; Fig. 4: pannels B, D.

## Data Availability

All data generated and analysed during this study are included in this published article and its additional information files.

## References

[1] Bernheim A, Sorek R (2020). The pan-immune system of bacteria: antiviral defence as a community resource. Nat Rev Microbiol..

[2] Koonin EV, Makarova KS, Wolf YI (2017). Evolutionary genomics of defense systems in archaea and bacteria. Annu Rev Microbiol.

[3] Hille F, Richter H, Wong SP, Bratovic M, Ressel S, Charpentier E (2018). The biology of CRISPR-Cas: backward and forward. Cell..

[4] van der Oost J, Westra ER, Jackson RN, Wiedenheft B (2014). Unravelling the structural and mechanistic basis of CRISPR-Cas systems. Nat Rev Microbiol.

[5] Nunez JK, Harrington LB, Kranzusch PJ, Engelman AN, Doudna JA (2015). Foreign DNA capture during CRISPR-Cas adaptive immunity. Nature..

[6] Nunez JK, Lee AS, Engelman A, Doudna JA (2015). Integrase-mediated spacer acquisition during CRISPR-Cas adaptive immunity. Nature..

[7] Nunez JK, Bai L, Harrington LB, Hinder TL, Doudna JA (2016). CRISPR immunological memory requires a host factor for specificity. Mol Cell.

[8] Jackson RN, Wiedenheft B (2015). A conserved structural chassis for mounting versatile CRISPR RNA-guided immune responses. Mol Cell.

[9] Makarova KS, Wolf YI, Alkhnbashi OS, Costa F, Shah SA, Saunders SJ (2015). An updated evolutionary classification of CRISPR-Cas systems. Nat Rev Microbiol..

[10] Makarova KS, Wolf YI, Iranzo J, Shmakov SA, Alkhnbashi OS, Brouns SJJ (2020). Evolutionary classification of CRISPR-Cas systems: a burst of class 2 and derived variants. Nat Rev Microbiol..

[11] Koonin EV, Makarova KS, Zhang F (2017). Diversity, classification and evolution of CRISPR-Cas systems. Curr Opin Microbiol.

[12] Yosef I, Manor M, Kiro R, Qimron U (2015). Temperate and lytic bacteriophages programmed to sensitize and kill antibiotic-resistant bacteria. Proc Natl Acad Sci U S A.

[13] Gomaa AA, Klumpe HE, Luo ML, Selle K, Barrangou R, Beisel CL (2014). Programmable removal of bacterial strains by use of genome-targeting CRISPR-Cas systems. MBio..

[14] Kiro R, Shitrit D, Qimron U (2014). Efficient engineering of a bacteriophage genome using the type I-E CRISPR-Cas system. RNA Biol.

[15] Box AM, McGuffie MJ, O'Hara BJ, Seed KD (2016). Functional analysis of bacteriophage immunity through a type I-E CRISPR-Cas system in Vibrio cholerae and its application in bacteriophage genome engineering. J Bacteriol.

[16] Vercoe RB, Chang JT, Dy RL, Taylor C, Gristwood T, Clulow JS (2013). Cytotoxic chromosomal targeting by CRISPR/Cas systems can reshape bacterial genomes and expel or remodel pathogenicity islands. PLoS Genet.

[17] Luo ML, Mullis AS, Leenay RT, Beisel CL (2015). Repurposing endogenous type I CRISPR-Cas systems for programmable gene repression. Nucleic Acids Res.

[18] Dolan AE, Hou Z, Xiao Y, Gramelspacher MJ, Heo J, Howden SE (2019). Introducing a spectrum of long-range genomic deletions in human embryonic stem cells using type I CRISPR-Cas. Mol Cell.

[19] Cameron P, Coons MM, Klompe SE, Lied AM, Smith SC, Vidal B (2019). Harnessing type I CRISPR-Cas systems for genome engineering in human cells. Nat Biotechnol.

[20] Young JK, Gasior SL, Jones S, Wang L, Navarro P, Vickroy B (2019). The repurposing of type I-E CRISPR-Cascade for gene activation in plants. Commun Biol.

[21] Morisaka H, Yoshimi K, Okuzaki Y, Gee P, Kunihiro Y, Sonpho E (2019). CRISPR-Cas3 induces broad and unidirectional genome editing in human cells. Nat Commun.

[22] Brouns SJ, Jore MM, Lundgren M, Westra ER, Slijkhuis RJ, Snijders AP (2008). Small CRISPR RNAs guide antiviral defense in prokaryotes. Science..

[23] Wiedenheft B, van Duijn E, Bultema JB, Waghmare SP, Zhou K, Barendregt A (2011). RNA-guided complex from a bacterial immune system enhances target recognition through seed sequence interactions. Proc Natl Acad Sci U S A.

[24] Deveau H, Barrangou R, Garneau JE, Labonte J, Fremaux C, Boyaval P (2008). Phage response to CRISPR-encoded resistance in *Streptococcus thermophilus*. J Bacteriol.

[25] Xue C, Zhu Y, Zhang X, Shin YK, Sashital DG (2017). Real-time observation of target search by the CRISPR surveillance complex Cascade. Cell Rep.

[26] Szczelkun MD, Tikhomirova MS, Sinkunas T, Gasiunas G, Karvelis T, Pschera P (2014). Direct observation of R-loop formation by single RNA-guided Cas9 and Cascade effector complexes. Proc Natl Acad Sci U S A.

[27] Hayes RP, Xiao Y, Ding F, van Erp PB, Rajashankar K, Bailey S (2016). Structural basis for promiscuous PAM recognition in type I-E Cascade from E. coli. Nature..

[28] Westra ER, van Erp PB, Kunne T, Wong SP, Staals RH, Seegers CL (2012). CRISPR immunity relies on the consecutive binding and degradation of negatively supercoiled invader DNA by Cascade and Cas3. Mol Cell.

[29] Rutkauskas M, Sinkunas T, Songailiene I, Tikhomirova MS, Siksnys V, Seidel R (2015). Directional R-loop formation by the CRISPR-Cas surveillance complex Cascade provides efficient off-target site rejection. Cell Rep.

[30] Hochstrasser ML, Taylor DW, Bhat P, Guegler CK, Sternberg SH, Nogales E (2014). CasA mediates Cas3-catalyzed target degradation during CRISPR RNA-guided interference. Proc Natl Acad Sci U S A.

[31] Semenova E, Jore MM, Datsenko KA, Semenova A, Westra ER, Wanner B (2011). Interference by clustered regularly interspaced short palindromic repeat (CRISPR) RNA is governed by a seed sequence. Proc Natl Acad Sci U S A.

[32] Xiao Y, Luo M, Hayes RP, Kim J, Ng S, Ding F (2017). Structure basis for directional R-loop formation and substrate handover mechanisms in type I CRISPR-Cas system. Cell..

[33] Xiao Y, Luo M, Dolan AE, Liao M, Ke A (2018). Structure basis for RNA-guided DNA degradation by Cascade and Cas3. Science.

[34] Sinkunas T, Gasiunas G, Waghmare SP, Dickman MJ, Barrangou R, Horvath P (2013). In vitro reconstitution of Cascade-mediated CRISPR immunity in Streptococcus thermophilus. EMBO J.

[35] Sinkunas T, Gasiunas G, Fremaux C, Barrangou R, Horvath P, Siksnys V (2011). Cas3 is a single-stranded DNA nuclease and ATP-dependent helicase in the CRISPR/Cas immune system. EMBO J.

[36] Loeff L, Brouns SJJ, Joo C (2018). Repetitive DNA reeling by the Cascade-Cas3 complex in nucleotide unwinding steps. Mol Cell.

[37] Fagerlund RD, Wilkinson ME, Klykov O, Barendregt A, Pearce FG, Kieper SN (2017). Spacer capture and integration by a type I-F Cas1-Cas2-3 CRISPR adaptation complex. Proc Natl Acad Sci U S A.

[38] Richter C, Gristwood T, Clulow JS, Fineran PC (2012). *In vivo* protein interactions and complex formation in the *Pectobacterium atrosepticum* subtype I-F CRISPR/Cas system. PLoS One.

[39] Rollins MF, Chowdhury S, Carter J, Golden SM, Wilkinson RA, Bondy-Denomy J (2017). Cas1 and the Csy complex are opposing regulators of Cas2/3 nuclease activity. Proc Natl Acad Sci U S A.

[40] Staals RH, Jackson SA, Biswas A, Brouns SJ, Brown CM, Fineran PC (2016). Interference-driven spacer acquisition is dominant over naive and primed adaptation in a native CRISPR-Cas system. Nat Commun.

[41] Richter C, Dy RL, McKenzie RE, Watson BN, Taylor C, Chang JT (2014). Priming in the type I-F CRISPR-Cas system triggers strand-independent spacer acquisition, bi-directionally from the primed protospacer. Nucleic Acids Res.

[42] Guo TW, Bartesaghi A, Yang H, Falconieri V, Rao P, Merk A (2017). Cryo-EM structures reveal mechanism and inhibition of DNA targeting by a CRISPR-Cas surveillance complex. Cell..

[43] Chowdhury S, Carter J, Rollins MF, Golden SM, Jackson RN, Hoffmann C (2017). Structure reveals mechanisms of viral suppressors that intercept a CRISPR RNA-guided surveillance complex. Cell..

[44] Mulepati S, Heroux A, Bailey S (2014). Crystal structure of a CRISPR RNA-guided surveillance complex bound to a ssDNA target. Science..

[45] Wiedenheft B, Lander GC, Zhou K, Jore MM, Brouns SJ, van der Oost J (2011). Structures of the RNA-guided surveillance complex from a bacterial immune system. Nature..

[46] Zhao H, Sheng G, Wang J, Wang M, Bunkoczi G, Gong W (2014). Crystal structure of the RNA-guided immune surveillance Cascade complex in *Escherichia coli*. Nature..

[47] Jorth P, Whiteley M (2012). An evolutionary link between natural transformation and CRISPR adaptive immunity. MBio..

[48] Crooks GE, Hon G, Chandonia JM, Brenner SE (2004). WebLogo: a sequence logo generator. Genome Res.

[49] Leenay RT, Maksimchuk KR, Slotkowski RA, Agrawal RN, Gomaa AA, Briner AE (2016). Identifying and visualizing functional PAM diversity across CRISPR-Cas systems. Mol Cell.

[50] Rollins MF, Schuman JT, Paulus K, Bukhari HS, Wiedenheft B (2015). Mechanism of foreign DNA recognition by a CRISPR RNA-guided surveillance complex from Pseudomonas aeruginosa. Nucleic Acids Res.

[51] Sternberg SH, Richter H, Charpentier E, Qimron U (2016). Adaptation in CRISPR-Cas systems. Mol Cell.

[52] Kuznedelov K, Mekler V, Lemak S, Tokmina-Lukaszewska M, Datsenko KA, Jain I (2016). Altered stoichiometry Escherichia coli Cascade complexes with shortened CRISPR RNA spacers are capable of interference and primed adaptation. Nucleic Acids Res.

[53] Kupczok A, Landan G, Dagan T (2015). The contribution of genetic recombination to CRISPR Array evolution. Genome Biol Evol.

[54] Klompe SE, Vo PLH, Halpin-Healy TS, Sternberg SH (2019). Transposon-encoded CRISPR-Cas systems direct RNA-guided DNA integration. Nature..

[55] Gleditzsch D, Muller-Esparza H, Pausch P, Sharma K, Dwarakanath S, Urlaub H (2016). Modulating the Cascade architecture of a minimal type I-F CRISPR-Cas system. Nucleic Acids Res.

[56] Jung C, Hawkins JA, Jones SK, Xiao Y, Rybarski JR, Dillard KE (2017). Massively parallel biophysical analysis of CRISPR-Cas complexes on next generation sequencing chips. Cell..

[57] Rollins MF, Chowdhury S, Carter J, Golden SM, Miettinen HM, Santiago-Frangos A (2019). Structure reveals a mechanism of CRISPR-RNA-guided nuclease recruitment and anti-CRISPR viral mimicry. Mol Cell.

[58] Pausch P, Muller-Esparza H, Gleditzsch D, Altegoer F, Randau L, Bange G (2017). Structural variation of type I-F CRISPR RNA guided DNA surveillance. Mol Cell.

[59] Luo ML, Jackson RN, Denny SR, Tokmina-Lukaszewska M, Maksimchuk KR, Lin W (2016). The CRISPR RNA-guided surveillance complex in Escherichia coli accommodates extended RNA spacers. Nucleic Acids Res.

[60] Songailiene I, Rutkauskas M, Sinkunas T, Manakova E, Wittig S, Schmidt C (2019). Decision-making in Cascade complexes harboring crRNAs of altered length. Cell Rep.

[61] Gu DH, Ha SC, Kim JS (2019). A CRISPR RNA is closely related with the size of the Cascade nucleoprotein complex. Front Microbiol.

[62] Drabavicius G, Sinkunas T, Silanskas A, Gasiunas G, Venclovas C, Siksnys V (2018). DnaQ exonuclease-like domain of Cas2 promotes spacer integration in a type I-E CRISPR-Cas system. EMBO Rep.

[63] Tamulaitis G, Mucke M, Siksnys V (2006). Biochemical and mutational analysis of EcoRII functional domains reveals evolutionary links between restriction enzymes. FEBS Lett.

