# Supplementary material for: DNA interference is controlled by R-loop length in a type I-F1 CRISPR-Cas system
Source: BMC Biol. 2020 Jun 15;18:65. doi: 10.1186/s12915-020-00799-z (PMC7296934; doi:10.1186/s12915-020-00799-z)
Supplement: Supplementary file 2 — Additional file 2: Figure S1. Cas2/3 nuclease and ATPase activity dependence on divalent metal ions. (A) Cas2/3-catalysed hydrolysis of ssDNA in the presence of different divalent metals. (B) Cas2/3-catalysed hydrolysis of ATP in the presence of different divalent metals. [file 12915_2020_799_MOESM2_ESM.pdf]

Additional file 2, Fig. S1

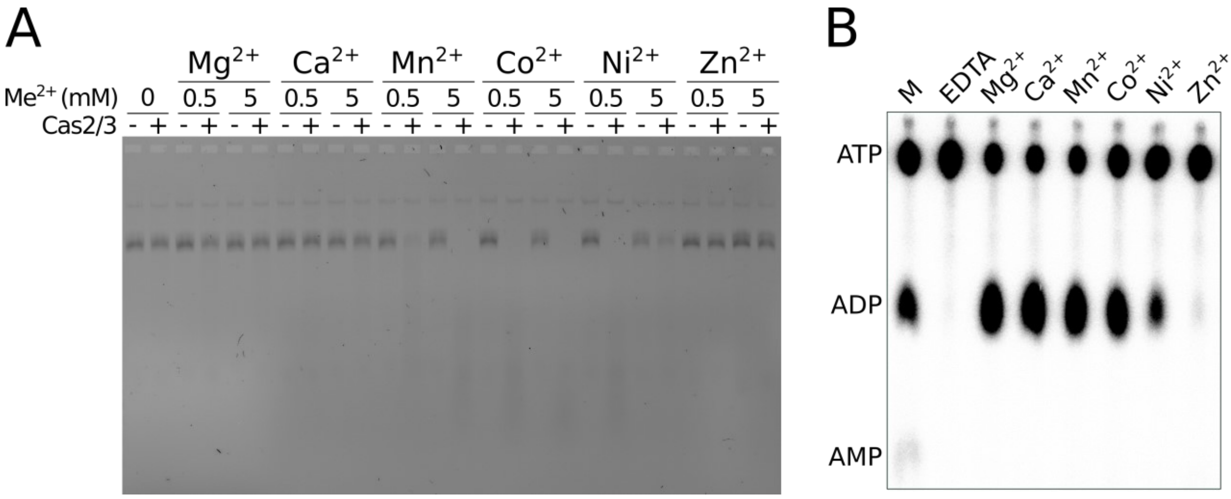

Fig. S1. *Cas2/3 nuclease and ATPase activity dependence on divalent metal ions.* (**A**) *Cas2/3-catalysed hydrolysis of ssDNA in the presence of different divalent metals*. Nuclease reactions were conducted at 37°C for 30 min in the N1 buffers containing 5 nM single-stranded circular M13mp18 DNA, 10 nM Cas2/3 and supplemented with 0.5 or 5 mM of indicated divalent metal ions. Reaction products were separated in agarose gel and visualised by SYBR Gold staining. (**B**) *Cas2/3-catalysed hydrolysis of ATP in the presence of different divalent metals*. ATPase reactions were conducted at 37°C for 20 min in the A buffers containing 2 mM [α^{32}P]-ATP, 5 nM M13mp18 ssDNA, 200 nM Cas2/3 and supplemented with 5 mM of indicated divalent metal ions or EDTA. Reaction products were separated on a thin-layer chromatography plate and visualized by phosphorimager.
