# Supplementary material for: DNA interference is controlled by R-loop length in a type I-F1 CRISPR-Cas system
Source: BMC Biol. 2020 Jun 15;18:65. doi: 10.1186/s12915-020-00799-z (PMC7296934; doi:10.1186/s12915-020-00799-z)
Supplement: Supplementary file 3 — Additional file 3: Figure S2. R-loop formed by WT Cascade. (A) Denaturing polyacrylamide gel of oligoduplex footprints. (B) R-loop within SP-CC oligoduplex sequence. [file 12915_2020_799_MOESM3_ESM.pdf]

Additional file 3, Fig. S2

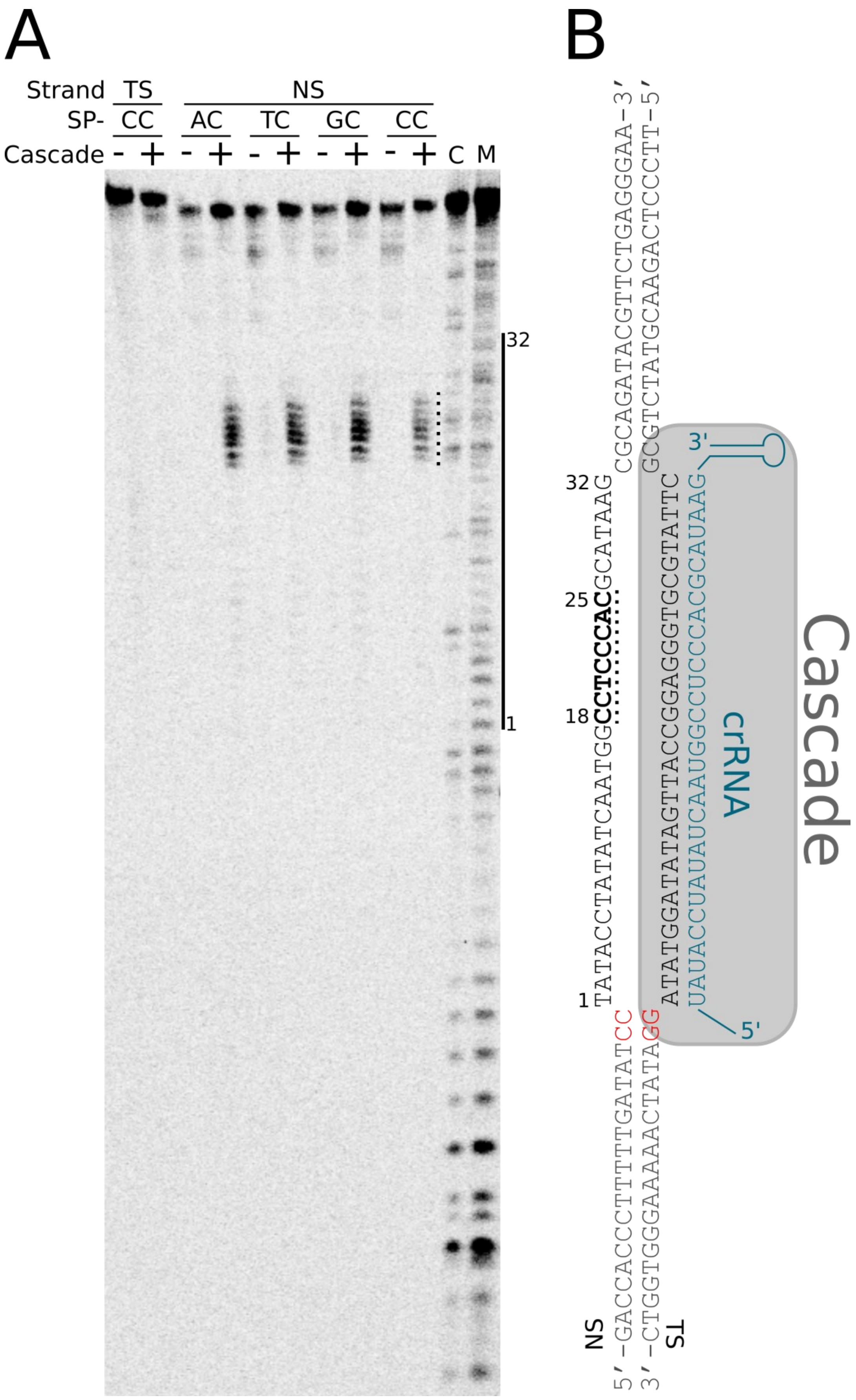

Fig. S2. *R-loop formed by WT Cascade.* (**A**)*. Denaturing polyacrylamide gel of oligoduplex footprints*. DNA oligoduplexes SP-CC, SP-AC, SP-GC, and SP-GC bearing the protospacer and PAM variant (CC, AC, GC, and GC, respectively) were ^{33}P-5´-labelled either on the target strand (TS) or on the non-target strand (NT) then treated with P1 nuclease in the absence (-) or presence (+) of WT Cascade complex. C and M represent ddCTP and mix of sequencing reactions of SP-CC non-target strand by ^{33}P-5´-labelled-oligonucleotide TS166, respectively. (**B**) *R-loop within SP-CC oligoduplex sequence*. R-loop is formed when crRNA of Cascade complex base-pairs with the target strand of the protospacer displacing the non-target strand as ssDNA, which is a substrate for P1 nuclease. A dashed line represents P1-sensitive region within 18th - 25th nt of the non-target strand of the protospacer. The protospacer region is indicated by a solid line and marginal nucleotides (1 and 32). The PAM is coloured in red.
