# Supplementary material for: DNA interference is controlled by R-loop length in a type I-F1 CRISPR-Cas system
Source: BMC Biol. 2020 Jun 15;18:65. doi: 10.1186/s12915-020-00799-z (PMC7296934; doi:10.1186/s12915-020-00799-z)
Supplement: Supplementary file 4 — Additional file 4: Figure S3. Cas2/3-mediated DNA degradation. (A) Preparation of linearized plasmid substrates. (B) Cas2/3 degradation of Cascade-bound linearized DNA targets. [file 12915_2020_799_MOESM4_ESM.pdf]

Additional file 4: Fig. S3

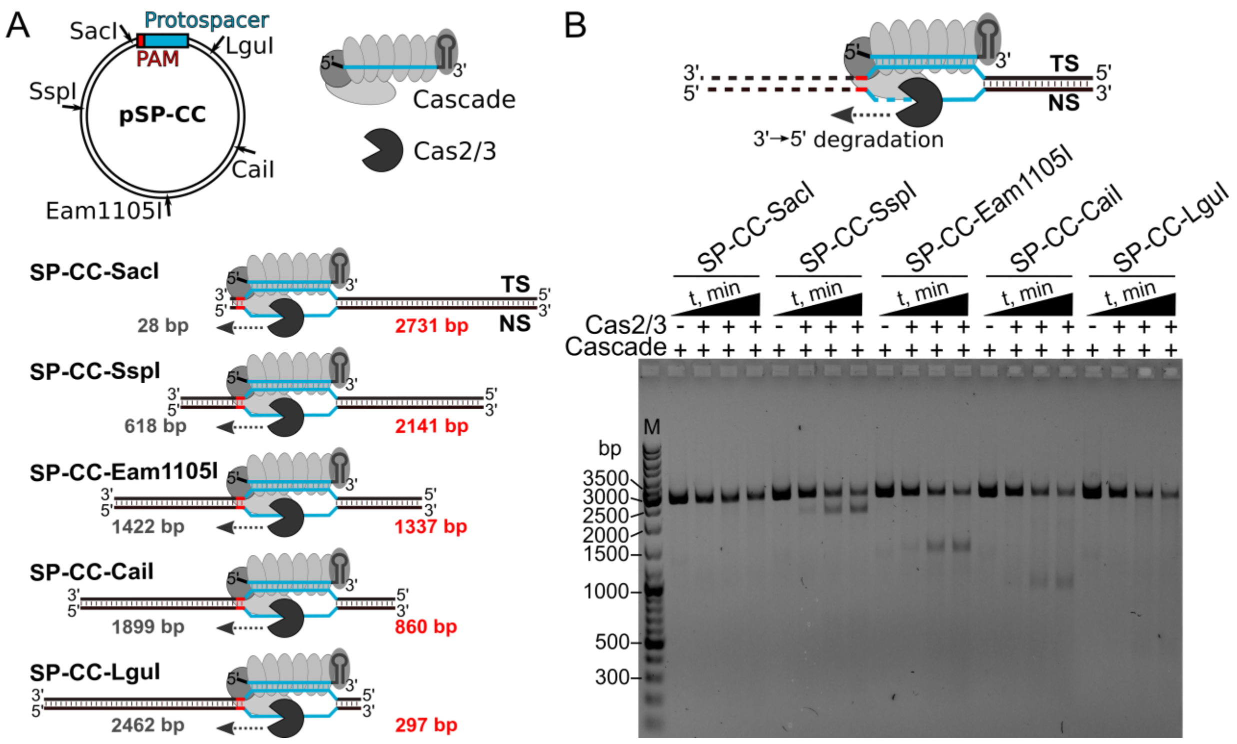

Fig. S3. *Cas2/3-mediated DNA degradation.* (**A**) *Preparation of linearized plasmid substrates*. Plasmid pSP-CC was cleaved using either of SacI, SspI, Eam1105I, CaiI, or LguI restriction endonucleases producing long (2759 bp) linear double-stranded DNA molecules that contain protospacer in different positions with the same directionality. A presumable double-stranded break between PAM and protospacer would generate DNA fragments of indicated lengths. (**B**) *Cas2/3 degradation of Cascade-bound linearized DNA targets*. Five linear DNA fragments pSP-CC-SacI, pSP-CC-SspI, pSP-CC-Eam1105I, pSP-CC-CaiI, and pSP-CC-LguI were pre-incubated with WT Cascade complex then treated with Cas2/3 (for 5, 30, and 60 min). Cas2/3 degrades DNA target upstream from the protospacer (in a protospacer-PAM direction) leaving downstream DNA fragment intact (red-coloured numbers in the **A**).
