# Supplementary material for: DNA interference is controlled by R-loop length in a type I-F1 CRISPR-Cas system
Source: BMC Biol. 2020 Jun 15;18:65. doi: 10.1186/s12915-020-00799-z (PMC7296934; doi:10.1186/s12915-020-00799-z)
Supplement: Supplementary file 5 — Additional file 5: Figure S4. Cas2/3 cleavage profile of Cascade-bound target dsDNA. Denaturing polyacrylamide gels of Cas2/3-cleaved oligoduplex labelled on either the non-target (A) or target (B) DNA strand. (C) Cleavage products mapped within the SP-CC oligoduplex sequence. [file 12915_2020_799_MOESM5_ESM.pdf]

Additional file 5, Fig. S4

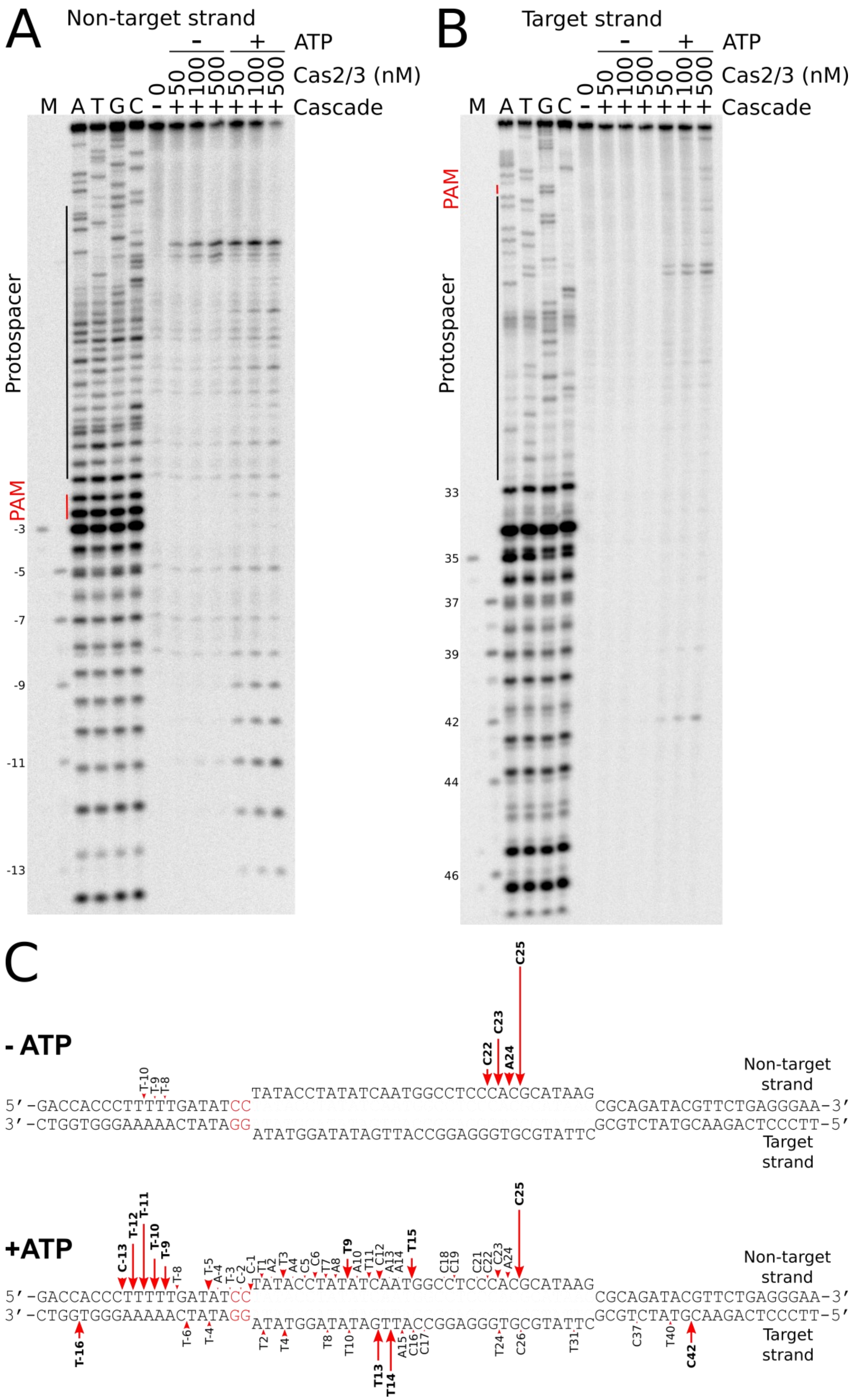

**Fig. S4**. *Cas2/3 cleavage profile of Cascade-bound target dsDNA.* (**A** and **B**) *Denaturing polyacrylamide gels of Cas2/3-cleaved oligoduplex.* SP-CC oligoduplex $^{32}$P-5´-labelled on either the non-target (**A**) or target (**B**) DNA strand was pre-incubated with WT Cascade in the absence (-) or presence (+) of ATP and cleavage was initiated by introducing indicated amounts of Cas2/3. Cleavage reactions were conducted at 37°C for 1 hour. A, T, G, C lanes represent sequencing reactions for the non-target and target strand of the SP-CC by the $^{32}$P-5´-labelled TS153 and TS166 oligonucleotides, respectively. M – ladders for smaller cleavage products (respective $^{32}$P-5´-labeled-oligonucleotides indicated in the Additional file 13: Table S3). Black and red solid lines designate protospacer and PAM boundaries, respectively. (**C**) *Cleavage products mapped within the SP-CC oligoduplex sequence*. Arrows indicate cleavage positions, the height of the arrow correlates with a relative amount of cleavage product after 1-hour incubation with 500 nM Cas2/3 in the absence (-ATP) or presence (+ATP) of ATP.
