# Supplementary material for: DNA interference is controlled by R-loop length in a type I-F1 CRISPR-Cas system
Source: BMC Biol. 2020 Jun 15;18:65. doi: 10.1186/s12915-020-00799-z (PMC7296934; doi:10.1186/s12915-020-00799-z)
Supplement: Supplementary file 6 — Additional file 6: Figure S5. R-loops formed by Cascade complexes containing truncated or extended spacers. Denaturing polyacrylamide gels of DNA duplex footprints after treatment with KMnO4 for a 60 s (A) or 20 s (B). (C) Cleavage pattern within the the non-target strand sequence. [file 12915_2020_799_MOESM6_ESM.pdf]

Additional file 6, Fig. S5

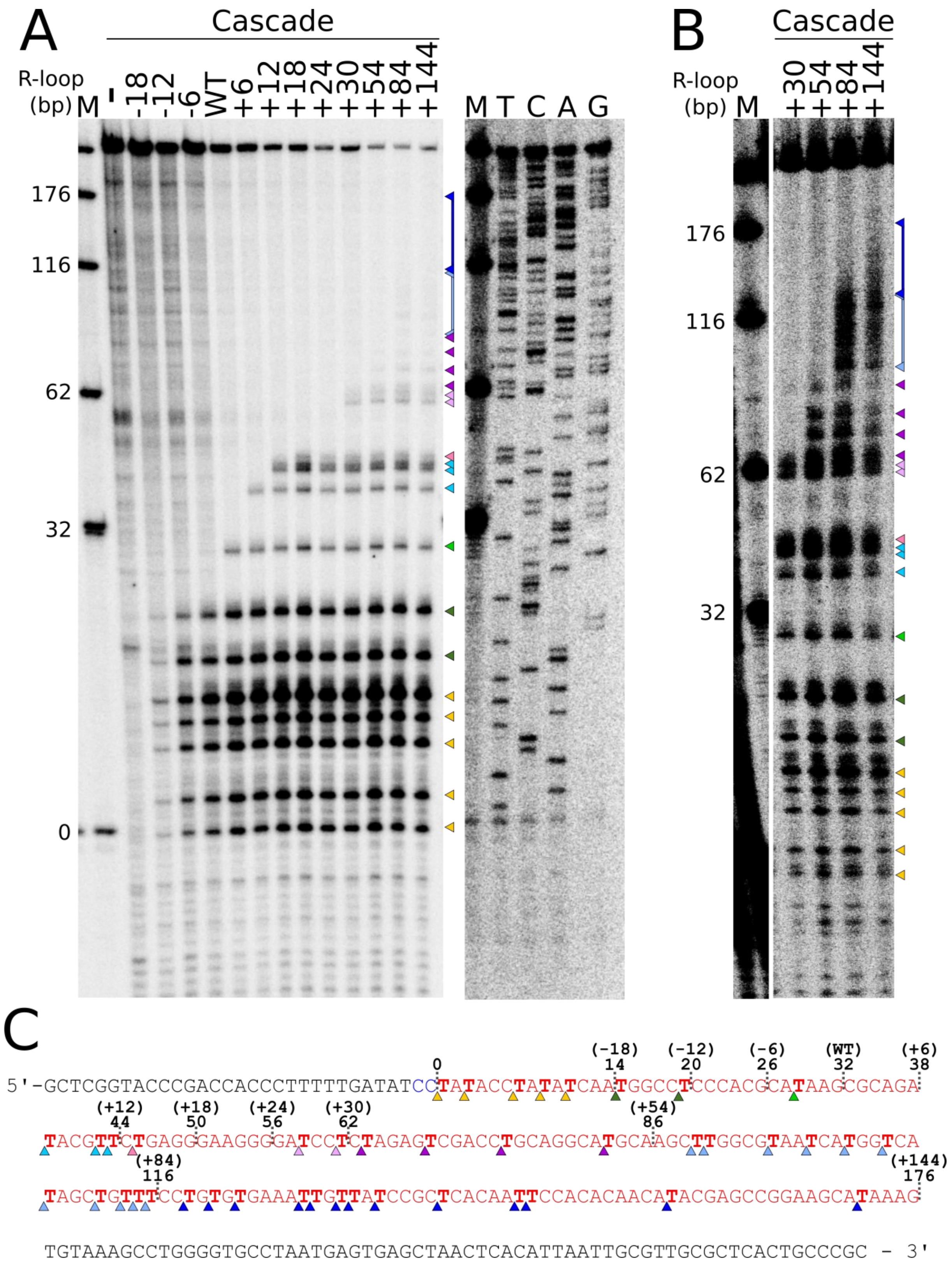

Fig. S5. *R-loops formed by Cascade complexes containing truncated or extended spacers.* (**A** and **B**) *Denaturing polyacrylamide gels of DNA duplex footprints*. DNA duplex SP-CC-3 was ^{32}P-labelled on the non-target DNA strand then DNA target was pre-incubated with a respective Cascade complex and treated with $KMnO_4$ for a 60 s (**A**) or 20 s (**B**). T, C, A, G lanes in the (**A**) represent sequencing

reactions for the non-targeting strand of the DNA target using TS911 ^{32}P-5´-labelled-oligonucleotide. The boundaries for the expected 32, 62, 116, and 176 bp length R-loops are indicated in the M lanes. (**C**) *The sequence of the non-target strand*. The protospacer region and PAM are coloured in red and blue, respectively. Triangles of different colours on the right of the gels (**A** and **B**) and within the non-target strand sequence (**C**) indicate cleavage positions near accessible thymidines (bold). Expected lengths of the R-loops formed by extended or truncated Cascade complexes (specified in parentheses) are indicated above the sequence.
