# Supplementary material for: DNA interference is controlled by R-loop length in a type I-F1 CRISPR-Cas system
Source: BMC Biol. 2020 Jun 15;18:65. doi: 10.1186/s12915-020-00799-z (PMC7296934; doi:10.1186/s12915-020-00799-z)
Supplement: Supplementary file 8 — Additional file 8: Figure S7. Cas2/3 cleavage of target DNA bound to truncated and extended Cascade complexes in the presence of ATP. Denaturing polyacrylamide gels of Cas2/3-cleaved DNA target labelled on either the non-target (A) or target (B) DNA strand. (C) Cleavage pattern within the DNA target. [file 12915_2020_799_MOESM8_ESM.pdf]

Additional file 8, Fig. S7

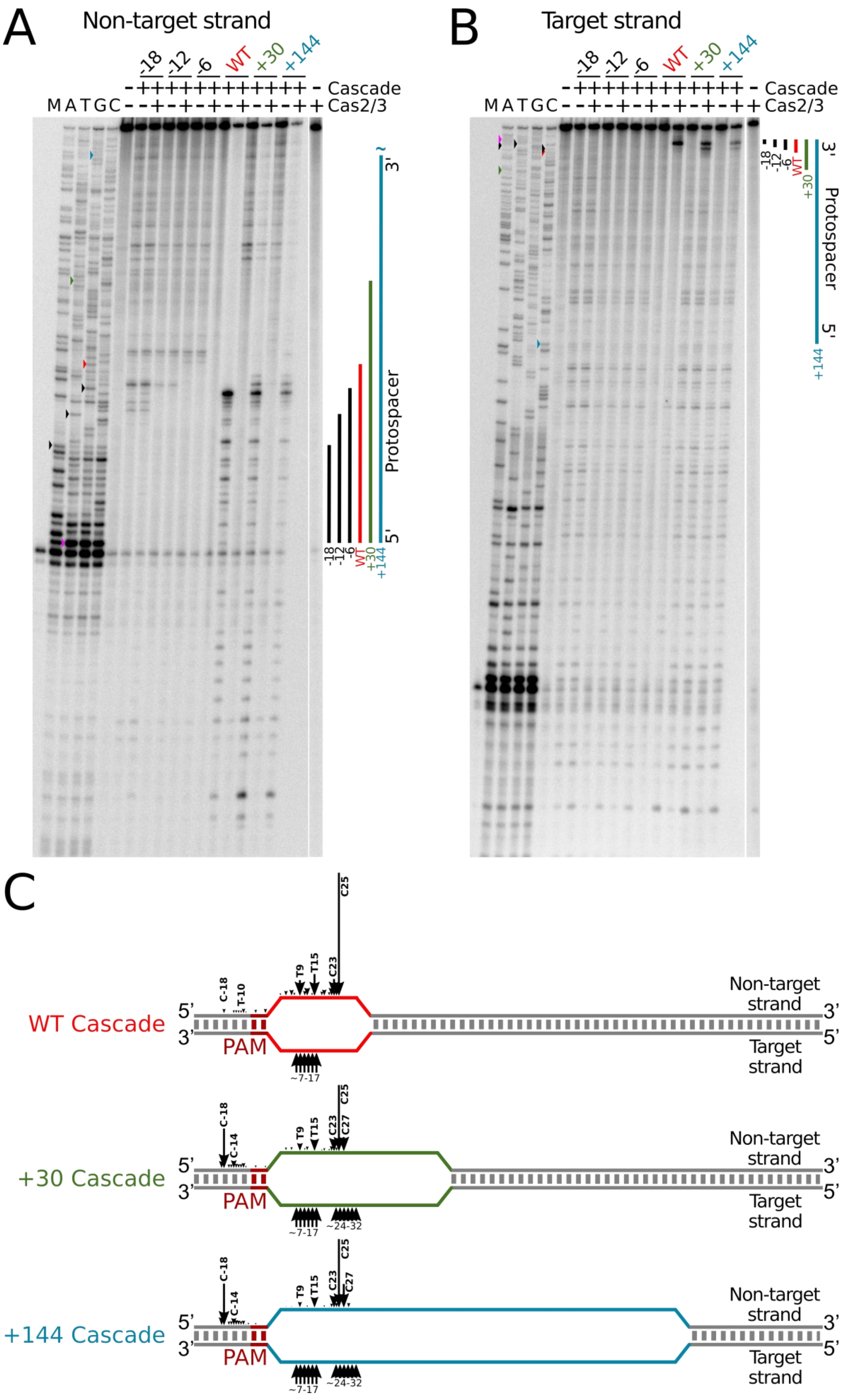

Fig. S7. *Cas2/3 cleavage of target DNA bound to truncated and extended Cascade complexes in the presence of ATP.* (**A** and **B**) *Denaturing polyacrylamide gels of Cas2/3-cleaved DNA target.* DNA duplex SP-CC-3 ^{32}P-labelled on either the non-target (**A**) or target (**B**) DNA strand was pre-incubated with the respective Cascade complex in the presence of ATP and cleavage was initiated by introducing the Cas2/3. T, C, A, G lanes represent sequencing reactions for the non-targeting and target strand of the SP-CC-3 using ^{32}P-5´-labelled TS911 and TS276 oligonucleotide (present in the M lane), respectively. Solid lines on the right of the gels indicate the protospacer region of the respective Cascade complex: red line represent WT Cascade complex, green and cyan – Cascade complexes with the extended spacers, while black lines show Cascade complexes with the truncated spacers that do not stimulate Cas2/3 cleavage. The purple triangle near the lanes of sequencing reactions specifies the first nucleotide in the protospacer counting from the PAM, while other triangles mark the last nucleotide of the protospacer in the respective R-loop. (**C**) *Cleavage pattern within the DNA target*. R-loops formed by WT and extended Cascade complexes are represented as bubbles of increasing length. Arrows indicate cleavage positions, the height of the arrow correlates with a relative amount of cleavage product after 1-hour incubation with 500 nM Cas2/3 in the presence of ATP. The major cleavage positions are assigned near the respective arrow.
