# Supplementary material for: DNA interference is controlled by R-loop length in a type I-F1 CRISPR-Cas system
Source: BMC Biol. 2020 Jun 15;18:65. doi: 10.1186/s12915-020-00799-z (PMC7296934; doi:10.1186/s12915-020-00799-z)
Supplement: Supplementary file 9 — Additional file 9: Figure S8. R-loops formed by WT and extended Cascade upon interaction with protospacers of different length. (A) Denaturing polyacrylamide gel of DNA duplex footprints. (B) Cleavage pattern within the non-target strand sequences. [file 12915_2020_799_MOESM9_ESM.pdf]

Additional file 9, Fig. S8

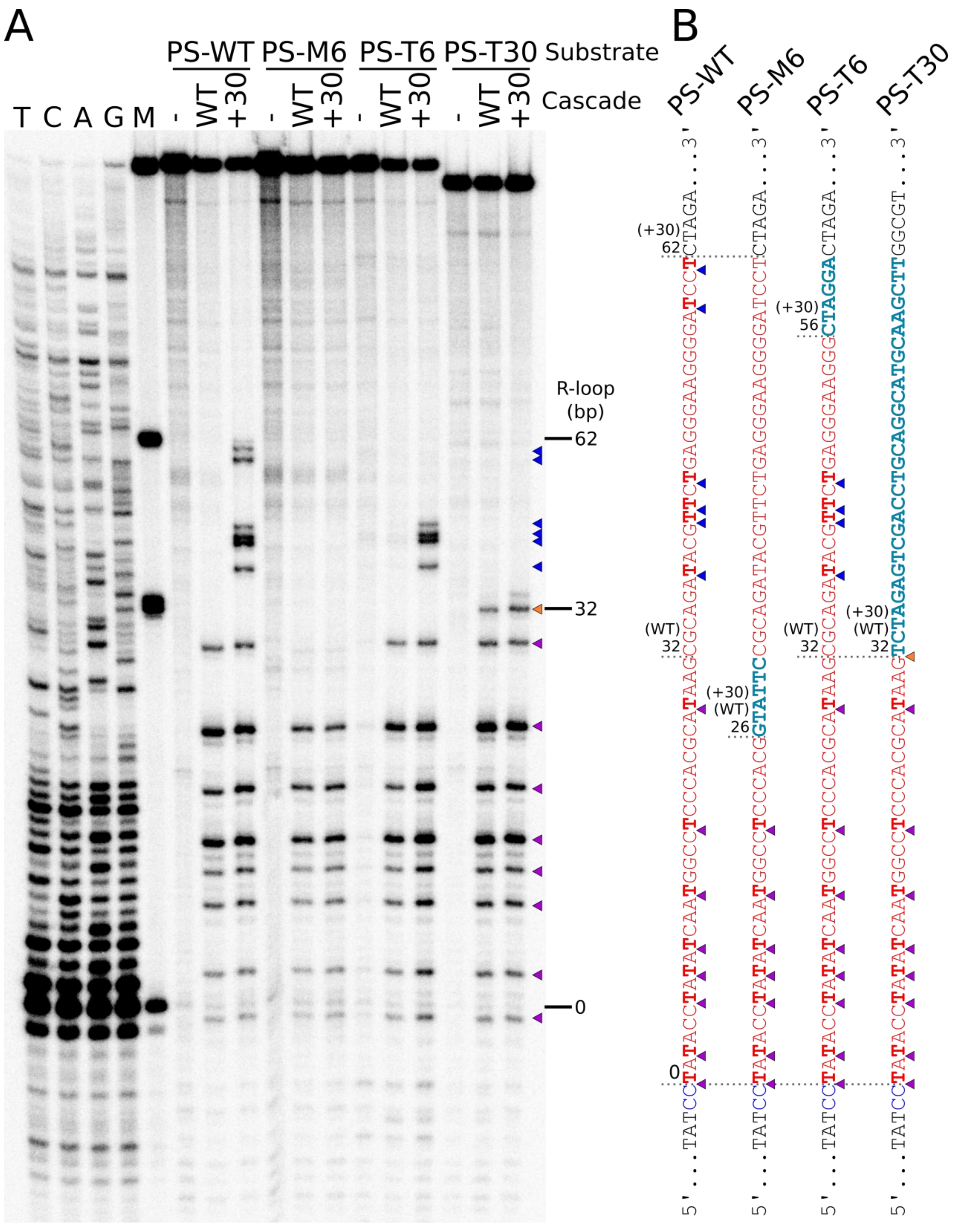

Fig. S8. R-loops formed by WT and extended Cascade upon interaction with protospacers of different length. (**A**) *Denaturing polyacrylamide gel of DNA duplex footprints*. DNA duplexes PS-WT, PS-M6, PS-T6 and PS-T30 were ^{32}P-labelled on the non-target DNA strand then pre-incubated with WT or +30 Cascade complex and treated with $KMnO_4$. T, C, A, G lanes represent sequencing reactions for the

non-target strand of the PS-WT duplex using ^{32}P-5´-labelled TS911 oligonucleotide. Boundaries for the expected 32 and 62 bp length R-loops are indicated in the M lanes. (**B**) *The sequence of the non-target strands*. The protospacer region and PAM are coloured in red and blue, respectively. Nucleotides mismatching the PS-WT protospacer are coloured in cyan. Triangles of different colours on the right of the gel (**A**) and within non-target strand sequences (**B**) indicate cleavage positions near accessible thymidines (bold). Expected lengths of the R-loops formed upon binding of WT or +30 Cascade complexes (specified in parentheses) to DNA target are indicated beside the sequences.
