# Supplementary material for: DNA interference is controlled by R-loop length in a type I-F1 CRISPR-Cas system
Source: BMC Biol. 2020 Jun 15;18:65. doi: 10.1186/s12915-020-00799-z (PMC7296934; doi:10.1186/s12915-020-00799-z)
Supplement: Supplementary file 10 — Additional file 10: Figure S9. Cas2/3 cleavage within protospacers of different length. Denaturing polyacrylamide gels of Cas2/3-cleaved DNA targets in the absence (A) or presence (B) of ATP. (C) Cleavage pattern within the non-target strand sequences. [file 12915_2020_799_MOESM10_ESM.pdf]

Additional file 10, Fig. S9

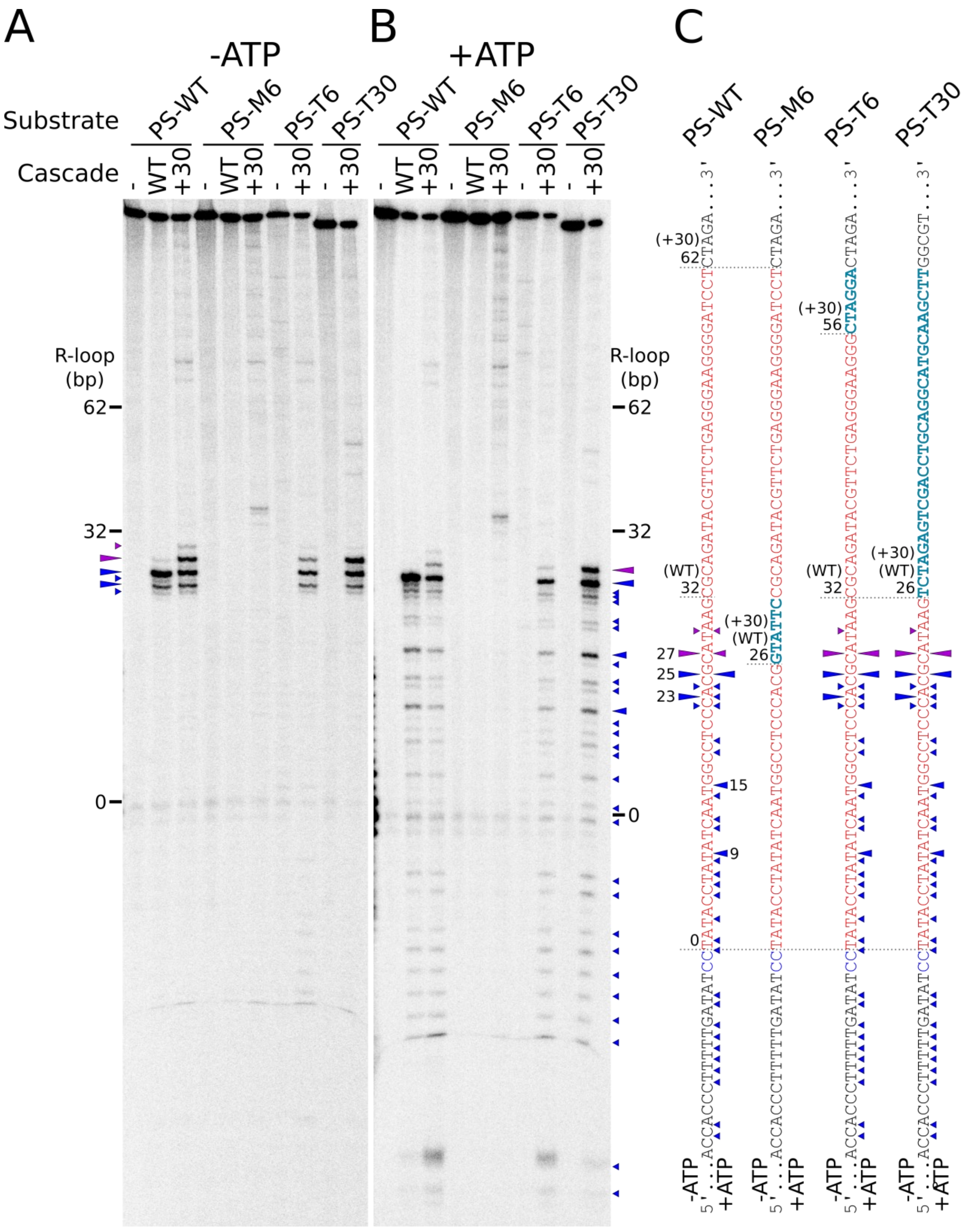

Fig. S9. *Cas2/3 cleavage within protospacers of different length.* (**A** and **B**) *Denaturing polyacrylamide gels of Cas2/3-cleaved DNA targets.* DNA duplexes PS-WT, PS-M6, PS-T6 and PS-T30 were ^{32}P-labelled on the non-target DNA strand then pre-incubated with WT or +30 Cascade complex in the absence (**A**) or presence (**B**) of ATP and cleavage was initiated by introducing the Cas2/3. The boundaries for the expected 32 and 62 bp length R-loops are indicated beside the gels. (**C**) *The*

sequence of the non-target strands. The protospacer region and PAM are coloured in red and blue, respectively. Nucleotides mismatching the PS-WT protospacer are coloured in cyan. Triangles of different colours on the right and left of the gels (**A** and **B**) and within non-target strand sequences (**C**) indicate cleavage positions of the Cas2/3. The height of the triangles correlates with a relative amount of cleavage product after 1-hour incubation with 500 nM Cas2/3 in the absence (-ATP) or presence (+ATP) of ATP. The expected lengths of the R-loops formed upon binding of WT or +30 Cascade complexes (specified in parentheses) to DNA target are indicated beside the sequences.
