# Supplementary material for: DNA interference is controlled by R-loop length in a type I-F1 CRISPR-Cas system
Source: BMC Biol. 2020 Jun 15;18:65. doi: 10.1186/s12915-020-00799-z (PMC7296934; doi:10.1186/s12915-020-00799-z)
Supplement: Supplementary file 11 — Additional file 11: Figure S10. Comparison of DNA interference stages in the type I-F1 and I-E CRISPR-Cas systems. (A) DNA interference mediated by the R-loop of WT length. (B) DNA interference mediated by the extended Cascade. (C) DNA interference mediated by the truncated Cascade. (D) Interference of DNA containing the truncated protospacer. (E) Interference of DNA containing a mismatch within the protospacer. [file 12915_2020_799_MOESM11_ESM.pdf]

Additional file 11, Fig. S10

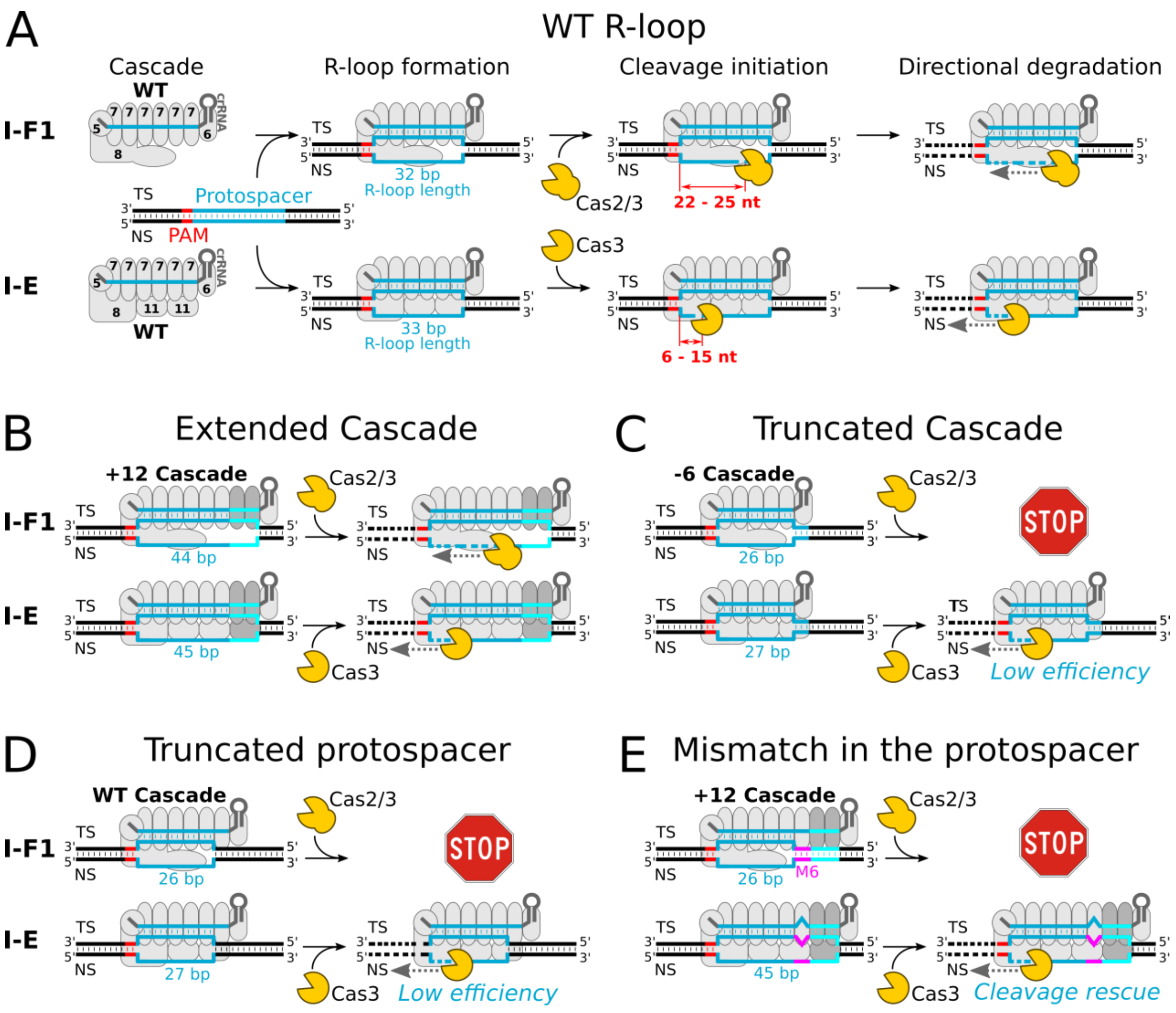

Fig. S10. *Comparison of DNA interference stages in the type I-F1 and I-E CRISPR-Cas systems.* (**A**) *DNA interference mediated by the R-loop of WT length.* Upon DNA target recognition, Cascade complexes (subunits numbered according Cas8 analogue, Cas5, Cas6 and Cas7 homologs or Cas11) form an R-loop (length is indicated below), where the spacer basepairs with the target strand (TS) of the protospacer displacing the non-target strand (NS). DNA cleavage in the type I-F1 and I-E systems is initiated at different sites: Cas2/3 cuts the NT at the PAM-distal end (22-25th nt), while Cas3 cleaves at the PAM-proximal end (6-15th nt), respectively. Then the Cas2/3 and Cas3 degrade DNA unidirectionally. (**B**) *DNA interference mediated by the extended Cascade.* The R-loops formed by the extended Cascades (e.g., +12) trigger DNA degradation similarly to the WT R-loops. (**C**) *DNA interference mediated by the truncated Cascade.* The R-loop formed by the truncated type I-F1 Cascade (e.g. -6) disables DNA cleavage initiation, while corresponding truncation of the type I-E Cascade maintains DNA cleavage although the efficiency is markedly reduced. (**D**) *Interference of DNA containing the truncated protospacer.* Binding of the WT or extended Cascade to the protospacers truncated at the PAM-distal end leads to the formation of shorter R-loop, which impairs DNA cleavage analogously to (**C**). (**E**) *Interference of DNA containing a mismatch within the protospacer.* Mismatch of 6 bp within the protospacer (M6; 27-32nd positions) disrupts base pairing of the extended type I-F1

Cascade (e.g. +12) leading to truncated R-loop (26 bp), which fails of DNA cleavage initiation. The extended type I-E Cascade overcomes analogous mismatch (28-33rd positions) forming full-length R-loop (45 bp), which is cleavaged with similar efficiency to WT R-loop. The extra Cascade subunits are coloured in darker grey, while WT, extended and mismatch (proto)spacer regions are bluish and cyan and magenta, respectively.
