# Supplementary material for: DNA interference is controlled by R-loop length in a type I-F1 CRISPR-Cas system
Source: BMC Biol. 2020 Jun 15;18:65. doi: 10.1186/s12915-020-00799-z (PMC7296934; doi:10.1186/s12915-020-00799-z)
Supplement: Supplementary file 12 — Additional file 12: Table S2. Expression vectors and plasmid substrates. [file 12915_2020_799_MOESM12_ESM.pdf]

Additional file 12, Table S2. Expression vectors and plasmid substrates.

Vector	Description	Cloning sites	Primers	Sorce
Expression				
pCDF-Duet1	T7 promoter based expression vector; Sm^R			Novagen
pCDF-SH	Derivative of pCDF-Duet1 where His_6 and S-tag sequences are replaced with Strep-II and His_6 sequences, respectively; Sm^R			[61]
pACYC-Duet1	T7 promoter based expression vector, Cm^R			Novagen
pCOLA-Duet1	*araC* promoter based expression vector, Kn^R			Novagen
pET-SH	Derivative of T7 promoter based expression vector pET-Duet1 where His_6 and S-tag sequences are replaced with Strep-II and His_6 sequences, respectively; Ap^R			[61]
pCd	*cas8f1-cas5f1-cas7f1-cas6f* cassete in pCDF-SH, no tags	NcoI/SalI	TS382/TS615	This study
pCd-H	*cas8f1-cas5f1-cas7f1* cassete and gene of Cas6f fused at N-terminus with His_6 sequence in pCDF-Duet1	NdeI/PacI	TS757/TS394	This study
		BamHI/SalI	TS397/TS758	
pCas1-2/3	*cas1-cas2/3* cassete in pCDF-SH, no tags	NdeI/PacI	TS616/TS417	This study
pCOLA-Cas1-2/3	*cas1-cas2/3* cassete in pCOLA-Duet1, no tags	NdeI/PacI	TS616/TS417	This study
pCas2/3-H	*cas2/3* gene in pET-SH; C-terminus of Cas2/3 is fused with His_6 tag	*NdeI/XhoI*	TS336/TS337	This study
pCd-Cas1-2/3	*cas1-cas2/3* cassete cloned into pCDF-Cd; no tag	*NdeI/PacI*	TS616/TS417	This study
pCd-Cas2/3	*cas2/3* gene cloned into pCDF-Cd; no tag	*NdeI/PacI*	TS336/TS417	This study
pCR-cloning	Repeats cloned into pACYC-Duet1	*NcoI/PacI*	TS543/TS544	This study
pCR(WT)	32 bp spacer cloned into pCR-cloning; spacer assembled from oligos	*SapI*	TS545/TS546	This study
pCR(-30)	2 bp spacer cloned into pCR-cloning; spacer assembled from oligos	*SapI/PacI*	TS772/TS773	This study
pCR(-24)	8 bp spacer cloned into pCR-cloning; spacer assembled from oligos	*SapI*	TS720/TS721	This study
pCR(-18)	14 bp spacer cloned into pCR-cloning; spacer assembled from oligos	*SapI*	TS718/TS719	This study
pCR(-12)	20 bp spacer cloned into pCR-cloning; spacer assembled from oligos	*SapI*	TS716/TS717	This study
pCR(-6)	26 bp spacer cloned into pCR-cloning; spacer assembled from oligos	*SapI*	TS714/TS715	This study
pCR(+6)	38 bp spacer cloned into pCR-cloning; spacer was PCR amplified using pSP-CC as template	*SapI*	TS723/TS724	This study
pCR(+12)	44 bp spacer cloned into pCR-cloning; spacer was PCR amplified using pSP-CC as template	*SapI*	TS723/TS725	This study

pCR(+18)	50 bp spacer cloned into pCR-cloning; spacer was PCR amplified using pSP-CC as template	*SapI*	TS723/TS726	This study
pCR(+24)	56 bp spacer cloned into pCR-cloning; spacer was PCR amplified using pSP-CC as template	*SapI*	TS723/TS727	This study
pCR(+30)	62 bp spacer cloned into pCR-cloning; spacer was PCR amplified using pSP-CC as template	*SapI*	TS723/TS728	This study
pCR(+54)	86 bp spacer cloned into pCR-cloning; spacer was PCR amplified using pSP-CC as template	*SapI*	TS723/TS829	This study
pCR(+84)	116 bp spacer cloned into pCR-cloning; spacer was PCR amplified using pSP-CC as template	*SapI*	TS723/TS830	This study
pCR(+144)	176 bp spacer cloned into pCR-cloning; spacer was PCR amplified using pSP-CC as template	*SapI*	TS723/TS776	This study
Substrates				
pSP-CC (also pPS-WT) [Target]	CC PAM and target protospacer sequences in pUC19 (pSP1-CC)			[34]
pNT [Non-target]	AA PAM and non-target protospacer sequences in pUC19 (pSP3-AA)			[34]
pSP-AA	AA PAM and target protospacer sequences in pUC19 (pSP1-AA)			[34]
pSP-AG	AG PAM and target protospacer sequences in pUC19 (pSP1-AG)			[34]
pSP-AC	AC PAM and target protospacer sequences in pUC19 (pSP1-AC)			[34]
pSP-AT	AT PAM and target protospacer sequences in pUC19 (pSP1-AT)			[34]
pSP-GA	GA PAM and target protospacer sequences in pUC19 (pSP1-GA)			[34]
pSP-GG	GG PAM and target protospacer sequences in pUC19 (pSP1-GG)			[34]
pSP-GC	GC PAM and target protospacer sequences in pUC19 (pSP1-GC)			[34]
pSP-GT	GT PAM and target protospacer sequences in pUC19 (pSP1-GT)			[34]
pSP-CA	CA PAM and target protospacer sequences in pUC19 (pSP1-CA)			[34]
pSP-CG	CG PAM and target protospacer sequences in pUC19 (pSP1-CG)			[34]
pSP-CT	CT PAM and target protospacer sequences in pUC19 (pSP1-CT)			[34]
pSP-TA	TA PAM and target protospacer sequences in pUC19 (pSP1-TA)			[34]
pSP-TG	TG PAM and target protospacer sequences in pUC19 (pSP1-TG)			[34]
pSP-TC	TC PAM and target protospacer sequences in pUC19 (pSP1-TC)			[34]
pSP-TT	TT PAM and target protospacer sequences in pUC19 (pSP1-TT)			[34]
pPS-T6	6 bp mutations was introduced within 57-62 positions from the CC		TS846/TS847	This study

	PAM in pSP-CC			
pPS-T30	Sequence within 33-62 positions from the CC PAM was deleted in pSP-CC		TS846/TS849	This study
pPS-M6	6 bp mutations was introduced within 27-32 positions from the CC PAM in pSP-CC		TS843/TS844	This study
M13mp18	ssDNA phage; ssDNA of this phage was used for helicase, nuclease and ATPase assays; replicative dsDNA was used for cloning procedures			NEB
M13-SP-CC [Target]	Target sequence was PCR amplefied from pSP-CC and cloned into M13mp18 phage	*EheI/BglI*	pUC-Ehe/TS911	This study
M13-NT [Non-target]	Non-target sequence was PCR amplefied from pNT and cloned into M13mp18 phage	*EheI/BglI*	pUC-Ehe/TS911	This study

REFERENCES

34. Sinkunas T, Gasiunas G, Waghmare SP, Dickman MJ, Barrangou R, Horvath P, Siksnys V: **In vitro reconstitution of Cascade-mediated CRISPR immunity in Streptococcus thermophilus**. *The EMBO journal* 2013, **32**(3):385-394.

61. Drabavicius G, Sinkunas T, Silanskas A, Gasiunas G, Venclovas C, Siksnys V: **DnaQ exonuclease-like domain of Cas2 promotes spacer integration in a type I-E CRISPR-Cas system**. *EMBO reports* 2018, **19**(7).
