# Supplementary material for: DNA interference is controlled by R-loop length in a type I-F1 CRISPR-Cas system
Source: BMC Biol. 2020 Jun 15;18:65. doi: 10.1186/s12915-020-00799-z (PMC7296934; doi:10.1186/s12915-020-00799-z)
Supplement: Supplementary file 13 — Additional file 13: Table S3. Oligonucleotides. [file 12915_2020_799_MOESM13_ESM.pdf]

Additional file 13, Table S3. Oligonucleotides.

Experiment	Subsrate name	Primer	Sequence (5´→3´)	Description
Cleavage, P1 footprints, Cloning	SP-CC	TS169	GACCACCCTTTTTGATATCCTATACCTATATCAATGGCCTCCCACGCATAAGCGCAGATACGTTCTGAGGGAA	Terget protospacer, CC PAM, ALQ13.2 phage surounding sequences
		TS170	TTCCCTCAGAACGTATCTGCGCTTATGCGTGGGAGGCCATTGATATAGGTATAGGATATCAAAAAGGGTGGTC	
	NT	TS140	GACCACCCTTTTTGATATAACGCAACCCCTCCTTAGACATGGGAACAGTACTAGCAGATACGTTCTGAGGGAA	Non-target protospacer, AA PAM, ALQ13.2 phage surounding sequences
		TS141	TTCCCTCAGAACGTATCTGCTAGTACTGTTCCCATGTCTAAGGAGGGGTTGCGTTATATCAAAAAGGGTGGTC	
	SP-AA	TS132	GACCACCCTTTTTGATATAATATACCTATATCAATGGCCTCCCACGCATAAGCGCAGATACGTTCTGAGGGAA	Target protospacer AA PAM, ALQ13.2 phage surounding sequences
		TS133	TTCCCTCAGAACGTATCTGCGCTTATGCGTGGGAGGCCATTGATATAGGTATATTATATCAAAAAGGGTGGTC	
EMSA	SP-CC-1	TS276	GTGATGACGGTGAAAACCTCTG	800 bp long oligoduplex with CC PAM and complementary protospacer; PCR amplified using pSP-CC as template.
		TS911	AAAAGCCATTCAGGCGCTGATACCGCTCGCCGCAG	
EMSA	NT-1	TS276	GTGATGACGGTGAAAACCTCTG	800 bp long oligoduplex without target sequence; PCR amplified using pNT as template
		TS911	AAAAGCCATTCAGGCGCTGATACCGCTCGCCGCAG	
EMSA	SP-AA-1	TS276	GTGATGACGGTGAAAACCTCTG	800 bp long oligoduplex with AA PAM and complementary protospacer, PCR amplified using pSP-AA as template
		TS911	AAAAGCCATTCAGGCGCTGATACCGCTCGCCGCAG	
EMSA	SP-CC-2	pUC57-Forw	GCCAGGGTTTTCCCAGTCACGA	420 bp long oligoduplex with CC PAM and complementary protospacer; PCR amplified using pSP-CC as template
		pUC-LguR	GCGAGGAAGCGGAAGAGCGCCC	
EMSA	SP3-AA-2	pUC57-Forw	GCCAGGGTTTTCCCAGTCACGA	420 bp long oligoduplex without target sequence; PCR amplified using pNT as template
		pUC-LguR	GCGAGGAAGCGGAAGAGCGCCC	
Cleavage, Footprinting	SP-CC-3 (also as PS-WT)	TS930[#]	GCGGGCAGTGAGCGCAACG	substrate for R-loop $KMnO_4$ footprinting and Cas2/3 cleavage assays; PCR amplified using pSP-CC as template
		TS931[*]	GCTCGGTACCCGACCACCCTTTTTGATATCC	
	PS-M6	TS930	GCGGGCAGTGAGCGCAACG	substrate for R-loop $KMnO_4$ footprinting and Cas2/3 cleavage assays; PCR amplified using pPS-M6 as template
		TS931[*]	GCTCGGTACCCGACCACCCTTTTTGATATCC	
	PS-T6	TS930	GCGGGCAGTGAGCGCAACG	substrate for R-loop $KMnO_4$ footprinting and Cas2/3 cleavage

		TS931*	GCTCGGTACCCGACCACCCTTTTTGATATCC	assays; PCR amplified using pPS-T6 as template
	PS-T30	TS930	GCGGGCAGTGAGCGCAACG	substrate for R-loop $KMnO_4$ footprinting and Cas2/3 cleavage assays; PCR amplified using pPS-T30 as template
		TS931*	GCTCGGTACCCGACCACCCTTTTTGATATCC	
Markers	M-32	TS932	CTTATGCGTGGGAGGCCAT	Marker for 32-bp R-loop boundary in $KMnO_4$ footprinting; PCR amplified using pSP-CC as template
		TS931*	GCTCGGTACCCGACCACCCTTTTTGATATCC	
	M-62	TS933	AGGATCCCCTTCCCTCAGA	Marker for 62-bp R-loop boundary in $KMnO_4$ footprinting; PCR amplified using pSP-CC as template
		TS931*	GCTCGGTACCCGACCACCCTTTTTGATATCC	
	M-116	TS934	AAACAGCTATGACCATGAT	Marker for 116-bp R-loop boundary in $KMnO_4$ footprinting; PCR amplified using pSP-CC as template
		TS931*	GCTCGGTACCCGACCACCCTTTTTGATATCC	
	M-176	TS935	CTTTATGCTTCCGGCTCGT	Marker for 176-bp R-loop boundary in $KMnO_4$ footprinting; PCR amplified using pSP-CC as template
		TS931*	GCTCGGTACCCGACCACCCTTTTTGATATCC	
		TS166	TTCCCTCAGAACGTATCT	^{32}P-5′-end-labelled oligonucleotides used for target strand dideoxy sequencing of SP-CC (complementary to TS169)
		TS153	GACCACCCTTTTTGATAT	^{32}P-5′-end-labelled oligonucleotides used for target strand dideoxy sequencing of SP-CC (complement to TS170)
		TS239	TTCCCTCAGAACGTAT	^{32}P-5′-end-labelled oligonucleotides used as markers (37, 39, 42, 44, and 46) for the target strand of the SP-CC.
		TS240	TTCCCTCAGAACGT	
		TS241	TTCCCTCAGAAC	
		TS242	TTCCCTCAGA	
		TS168	TTCCCTCA	
		TS232	GACCACCCTTTTTGAT	^{32}P-5′-end-labelled oligonucleotides used as markers (-5, -7, -9, -11, and -13) for the non-target strand of the SP-CC.
		TS233	GACCACCCTTTTTG	
		TS234	GACCACCCTTTT	
		TS235	GACCACCCTT	
		TS155	GACCACCC	
Cloning		TS336	t*catatg*aatatcttacttgttagccaatg	*cas2/3* + NdeI (fw)
		TS337	t*ctcgag*atcttcctgccaaaaacccagc	*cas2/3* + XhoI (rv)
		TS382	tatt*cgtctc*ccatgataactaaaacaacagtagtaaaagcc	*cas8f* + Esp3I (fw)
		TS394	gtgt*ttaattaa*ctattccttaccgcttgcgcc	*cas7* + PacI (rv)
		TS397	tatt*ggatcc*tatgacagtgcaaacccattacattg	*cas6f* + BamHI (fw)

		TS417	ag*ttaattaa*tcaatcttcctgccaaaaacccagc	*cas3* + PacI (rv)
		TS543	tat*ccatgg*cttcactgccgaataggcagcttagaaatgaagagccgagctcggcgcgcctgcaggtcgacaagc	pACYC-Duet1 + repeat + NcoI (fw)
		TS544	ctc*ttaattaa*tttctaagctgcctattcggcagtgaagtgaagagcgcttgtcgacctgcaggcgcgccgagctcg	pACYC-Duet1 + repeat + PacI (rv)
		TS545	aaatatacctatatcaatggcctcccacgcataag	32 bp (WT) spacer (fw)
		TS546	aagcttatgcgtgggaggccattgatataggtata	32 bp (WT) spacer (rv)
		TS615	tttt*gtcgac*ttagaaatgtggaacggttgtagtttg	*cas6f* + SalI (rv)
		TS616	tttt*catatg*cctaagacccccttacattc	*cas1* + NdeI (fw)
		TS714	aaatatacctatatcaatggcctcccacg	26 bp spacer (fw)
		TS715	aagcgtgggaggccattgatataggtata	26 bp spacer (rv)
		TS716	aaatatacctatatcaatggcct	20 bp spacer (fw)
		TS717	aagaggccattgatataggtata	20 bp spacer (rv)
		TS718	aaatatacctatatcaa	14 bp spacer (fw)
		TS719	aagttgatataggtata	14 bp spacer (rv)
		TS720	aaatatacctta	8 bp spacer (fw)
		TS721	aagtaggtata	8 bp spacer (rv)
		TS723	ata*gctcttc*aaaatatacctatatcaatggcctccc	elongated spacers + SapI (fw)
		TS724	ata*gctcttc*aaagtctgcgcttatgcgtggga	38 bp spacer + SapI (rv)
		TS725	ata*gctcttc*aaagaacgtatctgcgcttatgcg	44 bp spacer + SapI (rv)
		TS726	ata*gctcttc*aaagcctcagaacgtatctgcgc	50 bp spacer + SapI (rv)
		TS727	ata*gctcttc*aaagcccttccctcagaacgtat	56 bp spacer + SapI (rv)
		TS728	ata*gctcttc*aaagaggatccccttccctcaga	62 bp spacer + SapI (rv)
		TS757	tttt*catatg*ataactaaaacaacagtagtaaaagcc	*cas8f* + NdeI (fw)
		TS758	tttt*gtcgac*ttagaaatgtggaacggttgtagtttg	*cas6f* + SalI (rv)
		TS772	aaa*ta*cttcactgccgaataggcagcttagaaattaat	2bp spacer + repeat (fw)
		TS773	taatttctaagctgcctattcggcagtgaag*ta*	2bp spacer + repeat (rv)
		TS776	ata*gctcttc*aaagctttatgcttccggctcgtatg	176 bp spacer + SapI (rv)
		TS829	ata*gctcttc*aaagtgcatgcctgcaggtcgac	86 bp spacer + SapI (rv)

		TS830	ata*gctcttc*aaagaaacagctatgaccatgattacg	116 bp spacer + SapI (rv)
		TS843	GTATTCcgcagatacgttctgaggg	6 bp mismatch (within 27-32 bp positions) at the PAM distal end of the 32 bp protospacer (fw)
		TS844	cgtgggaggccattgatatag	6 bp mismatch (within 27-32 bp positions) at the PAM distal end of the 32 bp protospacer (rv)
		TS846	ctagagtcgacctgcaggc	6 bp mismatch and 30 bp deletion of the PAM distal end of the 62 bp protospacer (fw)
		TS847	TCCTAGcccttccctcagaacgtatc	6 bp mismatch (within 57-62 bp positions) at the PAM distal end of the 62 bp protospacer (rv)
		TS849	Acttatgcgtgggaggccattg	30 bp deletion (within 33-62 bp positions) at the PAM distal end of the 62 bp protospacer (rv)
		TS911	aaaa*gccattcaggc*gctgataccgctcgccgcag	pUC19 + BglI (rv); for cloning into M13mp18
		pUC-Ehe	ccgcatcaggcgccattcgcc	for cloning into M13mp18 (fw)

# ^{32}P-5′-end-labelled oligonucleotide was used for the DNA production with the labelled target strand;

* ^{32}P-5′-end-labelled oligonucleotide was used for the DNA production with the labelled non-target strand.
